# Economic shocks, health, and social protection: The effect of COVID‐19 income shocks on health and mitigation through cash transfers in South Africa

**DOI:** 10.1002/hec.4592

**Published:** 2022-08-23

**Authors:** Julius Ohrnberger

**Affiliations:** ^1^ School of Public Health Department of Infectious Disease Epidemiology Imperial College London St Mary's Campus London

**Keywords:** cash transfers, COVID‐19, difference‐in‐difference estimation, health, income shock, inequality, South Africa

## Abstract

COVID‐19 caused an unprecedented health and economic crisis. Nation‐wide lockdowns triggered major economic disruptions across the world. We provide evidence of the impact of these extreme economic shocks on health outcomes across wealth levels. We further identify if cash transfers can mitigate the negative health effects for the most economically vulnerable. The study focuses on South Africa, an Upper Middle‐Income Country with high levels of inequality, a large informal labor market and with low levels of social welfare. Using difference‐in‐difference estimation (DD) on a longitudinal sample of 6437 South Africans, we find that the lockdown income shock significantly reduces health by 0.2 standard deviations (SD). We find no difference of the effect across wealth quartiles. Exposure to a cash transfer program mitigates the negative health effects for recipients in the lowest wealth quartile to 0.25 SD compared to 0.4 SD for non‐recipients. Full mitigation occurs for individuals exposed to an on average higher scale‐up of the cash transfer program. Our analysis shows that a lockdown induced income shock caused adverse health outcomes; however, a pro‐poor cash transfer program protected the most economically vulnerable from these negative health effects.

## 1 INTRODUCTION

COVID‐19 related economic effects are devastating for vulnerable populations from Low‐ and Middle‐Income Countries (LMICs). COVID‐19‐suppression strategies like nation‐wide lockdowns led to huge disruptions of labor markets across the world, causing major income losses, especially among those more economically vulnerable (Jain et al., 2020; UNCTAD, 2020; WHO, 2020; Wills et al., 2020). There is a strong risk that these COVID‐19 lockdown induced income shocks exert strong negative effects on health outcomes (Bilal et al., 2017; Grossman, 1972; Gundersen & Ziliak, 2015; Jebena et al., 2017; Mwabu, 2007; Schiele & Schmitz, 2016; Strauss & Thomas, 1998; Wagstaff, 1986). Worse health outcomes can materialize due to lost health investment opportunities such as the inability to afford sufficient nutritious food as less disposable income is available (Strauss & Thomas, 1998). It is important to consider social safety nets like cash transfer programmes in this context, which can mitigate the supposedly negative health effects among vulnerable populations (Fiszbein & Schady, 2009; Gaarder et al., 2010; Porreca & Rosati, 2019; Skoufias & Di Maro, 2008; Skoufias et al., 2013). The focus of this paper is to provide first evidence of the average and distributional health effects resulting from a COVID‐19 lockdown induced income shock in South Africa. We further assess if a cash transfer program can mitigate the effect of the income shock on health for the economically vulnerable population.

The existing literature finds a negative relationship between income shocks and health outcomes. A systematic review by Margerison‐Zilko et al. (2016) shows that job‐loss during the financial crisis in 2007–2009 caused negative health effects such as increased morbidity, increased psychological distress and lower self‐rated health among populations from middle‐ and high‐income countries. A longitudinal study on the effects of the economic recession in Brazilian municipalities in 2014–2016 corroborates these findings in a LMIC‐setting, showing that recession‐related unemployment increased all‐cause mortality (Hone et al., 2019). Income shocks due to the loss of financial support following the premature death of a household member have further shown to negatively affect mental health outcomes among South African adults (Burger et al., 2017). The existing literature suggests that social support programmes can mitigate the negative health effects. Unemployment support programmes mitigated the negative health effects from the economic recession among European populations (Margerison‐Zilko et al., 2016). Similarly, Hone et al. (2019) find no significant associations of recession‐related unemployment increased all‐cause mortality in Brazilian municipalities with high social protection expenditures.

A limitation of existing research on the income‐shock and health relationship is that they do not provide an understanding of the heterogenous distributional health effects which are important to understand to target policies. Another limitation is that studies lack robust causal methods to establish the causal impact of both the income shock on health and the mitigation effect of social protection programmes. Neither provide existing studies an understanding of the scale‐up effects of social support programmes on health effects. The context of the analyzed recession and bereavement related income shocks are also very different to the COVID‐19 lockdown induce income shocks. The latter are unprecedented in their magnitude, their scope, and in their abruptness in modern times (ILO‐OECD, 2020; World Bank, 2020). The immediate decline of the working population, following national lockdowns across the world in April 2020, exceeded the 2‐year effects of the financial crisis 2007–2009 by 14 times, affecting all national and global supply‐chains and different economic sectors suddenly and simultaneously (ILO‐OECD, 2020). The severity of the shock may affect economically more vulnerable populations disproportionately which emphasizes the importance to understand the distributional health effects and how cash transfer programmes mitigate the effects for this population.

We aim to fill these gaps in the literature by understanding the effect of COVID‐19 lockdown induced income‐shocks on individual health among populations living in South Africa. Our first objective is to identify the causal effect of the lockdown‐induced income loss on individual health. The second objective is to understand the distributional health effects of the income shock by wealth quartiles. Our third objective is to establish the mitigation effects of a scaled‐up cash transfer program on health for the those in the lowest wealth quartile, that is, the most economically vulnerable population. Building on the existing evidence and theory, we hypothesize negative health effects due to the income‐shock across the wealth distribution with marginally lower effects for individuals protected by the cash transfer. We further contribute to the literature by using unique longitudinal data which provides individual observations across a time span of 12 years. The rich longitudinal data gives us the opportunity to use robust causal inference methods. This is of importance for research focusing on LMICs where such data is scarce and causal inference often difficult to establish.

We focus in the analysis on South Africa, as the country has experienced the highest prevalence of COVID‐19 infections in Sub‐Saharan Africa and has also seen a significant disruption in economic activities (Drain & Garrett, 2020). In a major response to the COVID‐19 induced economic crisis, the South African government scaled‐up the country's largest cash transfer program, the Child Support Grant (CSG) (Köhler & Bhorat, 2020). This provides us with an ideal setting to explore different variations of a large‐scale social protection program and related consequences for health in response to the lockdown induced income shock. We argue that lockdown effects on health outcomes are driven by income shocks. This makes sense in a country like South Africa which has a young labor force with 90% in the labor force being under age 55 and the pre‐delta variant strain of COVID‐19 causing mostly asymptomatic cases among the population below age 60 (Statistics South Africa (SSA), 2020b; Poletti et al., 2021). National data on employment supports this argument as most individuals report to have stopped working for the lockdown itself and not due to health related reasons (Statistics South Africa (SSA), 2020b).

We build our analysis on the first wave of the National Income Dynamics Study – Coronavirus Rapid Mobile Survey (NIDS‐CRAM). The NIDS‐CRAM is a broadly nationally representative panel study of South African adults of age 18 and older (Ingle et al., 2020). We supplement the analysis with data from five waves of the National Income Dynamics Study (NIDS) ranging from 2008 until 2018 (Leibbrandt et al., 2009). Our sample is comprised of 6437 individuals who are observed in both the NIDS‐CRAM and the NIDS. Individual health is measured by self‐rated health and lockdown induced income shock is measured by the loss of the main source of income at the household level. To identify the health effects of exposure to the income shock, we use difference‐in‐difference (DD) estimation. We then use a heterogeneous difference‐in‐difference analysis to understand, firstly, if health effects vary by wealth quartiles, and secondly, if the CSG mitigated the hypothesized negative health effects in the lowest wealth quartile.

## 2 CONTEXT

The first South African COVID‐19 case was detected on March 5^th^, 2020. Toward the end of the month, 1353 cases were reported (44,292 tests), with five COVID‐19 related deaths. These numbers further increased to 32,683 cases (725,125 tests) and 638 deaths toward the end of May. By mid‐June 2020, 61,927 cases (1,060,425 tests) and 1354 COVID‐19 related deaths were reported (Mbunge, 2020). The first COVID‐19 wave peaked in‐July 2020 in South Africa (Salyer et al., 2021). In response to the COVID‐19 outbreak and the growing pressure on the health system, the South African government established a hard national lockdown on March 27, 2020. It restricted economic activities to essential services, limited public transport significantly, and forbid inter‐provincial journeys. The lockdown was first adjusted on April 30, 2020, permitting economic activities in certain sectors (i.e., mining, agriculture, and finance) and essential services. Restrictions on transport and personal freedom of movement were kept in place. A further adjustment to the lockdown was made at the beginning of June, permitting the retail business to reopen. Public transport and the freedom of movement remained restricted (Department of Health South Africa, 2021; South African Government, 2021).

The economic consequences of the national lockdown were immense for the South African economy. Unemployment in both the informal and formal sector rose significantly by 2.2 million people in the months following the country's national lockdown (Haider et al., 2020; Jain et al., 2020). This resulted in large lockdown related wage‐income losses, especially among low‐skilled populations in South Africa, putting the food security of low‐income households at risk (Arndt et al., 2020). Whilst all sectors of the South African economy recorded losses in employment in the second quarter of 2020, the hardest hit sectors were finance and business, construction, manufacturing, services, and trade which account for more than 50% of the South African economy (Statistics South Africa (SSA), 2020a). Parts of the labor force could mitigate the negative effects of the lockdown on their economic activity by shifting to a working from home routine. However, this ability was limited and possibly further increased income inequalities in the country as evidence from non‐white populations, populations living in informal settlements and in urban areas and individuals engaged in the formal sector, especially in services, mining and manufacturing were less likely to be able to work from home (Benhura & Magejo, 2021). The lockdown included income shock showed early signs of limited health investment possibilities. Findings from a study using nationally representative data show that about half of the interviewed South Africans reported insufficient funds to buy food in April 2020. About 20% reported to have gone hungry to bed in the months of May and June in 2020 (Wills et al., 2020).

## 3 DATA

### 3.1 Data sources

We combine two sources of data. The main source is the first wave of the NIDS‐CRAM. The NIDS‐CRAM is a nationally representative panel survey of South African individuals of age 18 and older and provides information of COVID‐19 related impacts on health, socio‐economic outcomes and social support (Ingle et al., 2020). The NIDS‐CRAM is set to be conducted as a panel study every month from May until October 2020 using phone interviews of one adult per household. Data collection of the first wave was conducted in May and June 2020, about two months since the COVID‐19 lockdown was imposed in South Africa. As such the first wave of the NIDS‐CRAM provides us with ideal data to understand the short‐term impacts of lockdown related income shocks on health. The study sample was derived from the latest wave of the NIDS sample in 2017/2018. A stratified sampling design with batch sampling was used, randomly selecting a household member (Kerr et al., 2020). Batch‐sampling splits the targeted population into small batches, starting with one initial batch. Strata sampling rates are adjusted in the following batches according to response rates (i.e., over‐ or under‐sampling in the strata in the subsequent batches depending on the initial responses). Batch sampling permits to adjust for non‐responsiveness during the data collection.

The second data source is the NIDS, a representative biennial longitudinal study of the South African population which provides information on socio‐economic status, household variables, health outcomes and neighborhood characteristics (Leibbrandt et al., 2009). We use in the analysis all available five waves of the NIDS, from 2008 until 2018. Our sample of 6437 individuals is observed in various waves of the NIDS and constitutes an unbalanced panel. Due to the design of the NIDS‐CRAM, which is drawn from the NIDS sample in 2017/2018, all individuals in our data are observed in 2020 and in 2017/2018, but their number of observations in the other pre‐treatment period varies. This is as individuals from the NIDS sample in 2017/18 can enter the NIDS at various points in time; firstly, by being part of the initial panel in 2008, secondly, by moving into an existing NIDS household at any given time in the pre‐intervention period, or thirdly becoming age‐eligible for the adult surveys (age 15+). Table A1 in the supplementary material presents the evolution of the panel over time, showing that most individuals of the NIDS‐CRAM sample joined the NIDS panel survey in 2008 (4012 individual or 62% of the sample), which is the first wave of the NIDS. Combining both datasets enables us to use and test our identification strategy of DD and the heterogeneous DD.

### 3.2 The income shock

We can make a strong case that observed changes in health due to income losses are driven by the lockdown, not individual health problems, health problems of household members or health preferences related to COVID‐19 infection or the risk of an infection.

**FIGURE 1:**
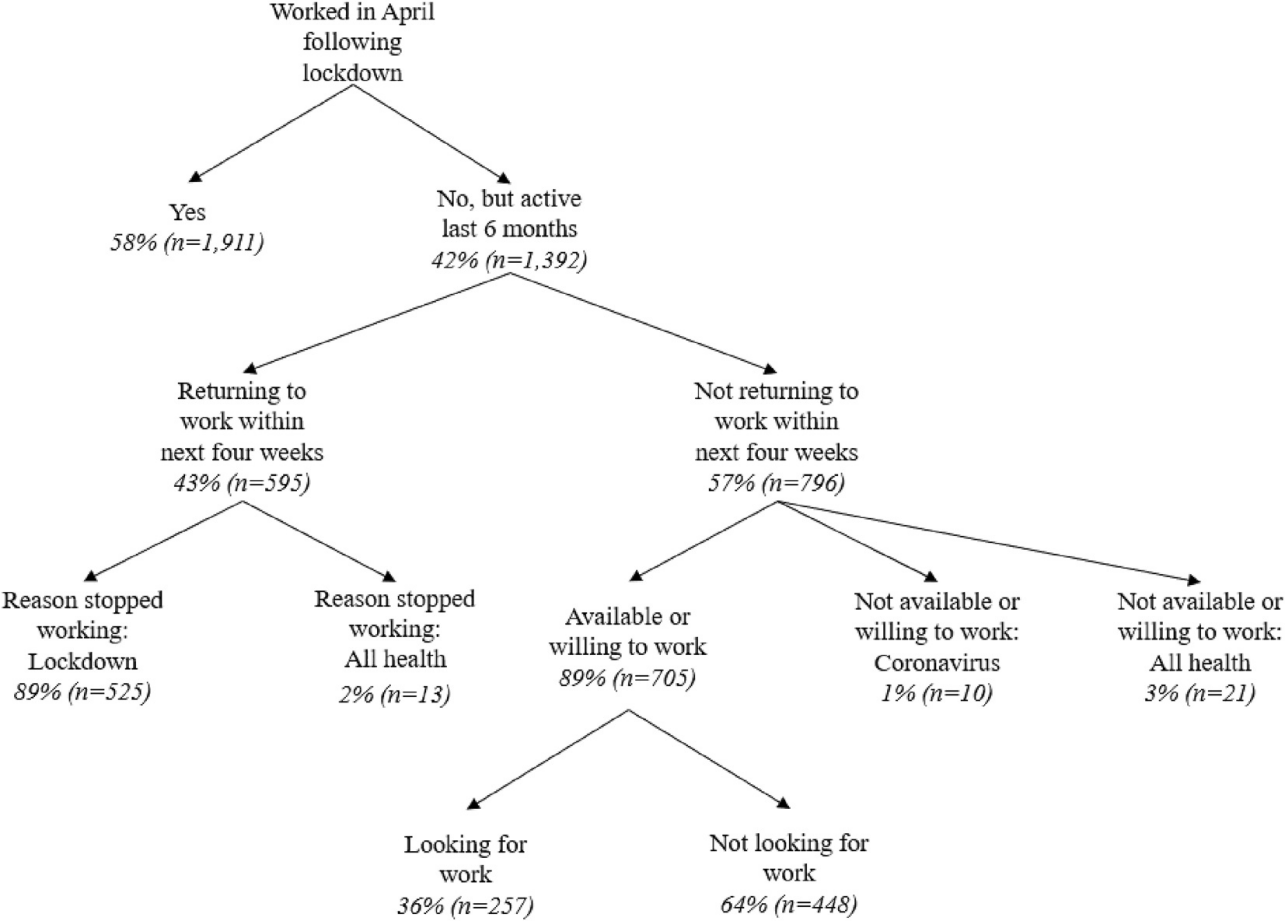
Evolution of work patterns during the lockdown. Descriptive work characteristics on the estimation sample, using data from the first wave of the National Income Dynamics Study – Coronavirus Rapid Mobile Survey (NIDS‐CRAM). Statistics are presented for individuals that were either economically active during the lockdown or have not been active during the lockdown but were economically active over the course of the last six months prior to the interview; † includes all health reasons such as ill health, caring for ill family member or more specifically COVID‐19 related reasons

Figure 1 illustrates work patterns for economically active individuals within 6 months prior to the interview in the first wave of the NIDS‐CRAM. Information for economically inactive individuals is not available in the NIDS‐CRAM; however, those individuals will be unaffected by lockdown or health reasons due to their inactive economics status irrespective of the COVID‐19 pandemic. 42% of the sample were not economically active during the lockdown. About half of those state their intention to return to work within the next 4 weeks. 89% of those state that the lockdown itself and not health reasons caused them to stop working. Less than one percent of those returning to work within the next four weeks state explicitly COVID‐19 as reason for stopping to work. Among those economically active prior to the lockdown but not planning to return to work within the next four weeks, only 1% state that COVID‐19 related reasons stop them from returning to work. This clearly illustrates that the lockdown caused the economic disruptions for those individuals who were only temporarily affected in their economic activities, not COVID‐19 or other health reasons.

Data from Quarterly Labor Force Surveys in South Africa from 2015 to 2020 further confirms the abnormal temporary impact of the lockdown on economic activity. Total employment (including both formal and informal) dropped by 2.2 million in the second quarter of 2020, which is the period immediately affected by the lockdown, compared to the first quarter in 2020 which is unaffected by the lockdown. This is a huge change in employment compared to previous changes in the preceding five years (2015, 2016, 2017, 2018, 2019) which range between decreases in employment of 129,000 and increases of 198,000. Importantly, about 80% of those individuals temporarily out of work in the second quarter of 2020 report that the lockdown itself is the main reason preventing them from working (Statistics South Africa (SSA), 2021).

### 3.3 Variables

#### 3.3.1 Health outcome measure

We measure individual health with a self‐rated health variable. The respondents were asked whether s/he would rate his/her health at present as 0 “poor”, 1 “fair”, 2 “good”, 3“very good”, or 4 “excellent”. Self‐rated health has been used in previous analysis and is considered a good measure of general health status across time,encompassing both mental and physical health components (Beck et al., 2015; Contoyannis & Jones, 2004; Huijts et al., 2015; Kim, 2015; Pega et al., 2017).

#### 3.3.2 Exposure measure

Our exposure measure or treatment indicator is a binary variable taking value one if the individual reported to live in a household which has lost the main source of income since the beginning of the lockdown on 27 March and is zero otherwise. The interviewed individual or another household member can account for the loss of the main source of household income. As such exposure is identified on the household‐level and not constraint by the economic activity of the reporting individual.

#### 3.3.3 Wealth quartiles measure

The NIDS provides information on the total value of assets of a household in South African Rand. The measure is summed over the value of real estate, business, and financial assets, vehicles, retirement annuities, livestock and durable household goods (Daniels & Khan, 2019). We compute asset quartiles using values from 2017/2018, preceding the income shock. Thusly, the wealth measure is independent of the shock. This is important, as we aim to understand to what extend the shock affected the populations that were vulnerable prior the lockdown. We first adjust the total value of household assets in South African Rand for inflation using monthly consumer price index values with baseline in December 2016. We then compute the inflation adjusted per capita household total assets using the squared‐root method by dividing the real total assets with the squared‐root of household members and compute the quartiles subsequently (Dudel et al., 2021; OECD, 2011). Assets are widely regarded as strong measure for household wealth and usually computed over an asset index of durable items owned by the household (O’Donnell et al., 2008). We have the advantage of real financial values which adds more precision in identifying the wealth‐level of a household. A previous analysis further found that purely durable goods based assets have poor internal validity among the NIDS study sample (Wittenberg & Leibbrandt, 2017).

#### 3.3.4 Cash transfer measure

We utilize information of household receipt of South Africa's largest cash transfer program, the Child Support Grant (CSG). The CSG is discussed in detail elsewhere (UNICEF, DSD and SASSA, 2012). The CSG is targeted to improve the livelihoods of children living in poverty in South Africa. It is a means targeted anti‐poverty program. Care takers of a child/children can apply to receive the monthly cash transfer on behalf of their child (ren). Existing research shows that the CSG is a substantial income source for receiving households, of about 20%–25% of the household income, which is commonly shared between household members. It is thus affecting all household members (Cluver et al., 2013; Delaney et al., 2008; Gomersall, 2013).

In response to COVID‐19 and the economic hardship of the lockdown, the South African government scaled the CSG up at the intensive margin (Köhler & Bhorat, 2020). In May 2020, the government increased the CSG by an additional R300 per child, a 70% increase from the initial amount of R440 per child. From June onwards, the scale‐up changed to R500 per care giver only. The cash transfer amount is larger for households with more than one receiving child in May and it is larger in June for households with only one receiving child. Our data suggests that 1.5 children per CSG‐receiving household receive the CSG in 2020, with on average two children in the lowest wealth quartile. These numbers are comparable between individuals exposed and unexposed to the shock.

We use two types of self‐reported CSG variables. Firstly, a binary variable indicating if the individual lives in a CSG‐receiving household in 2020 (No CSG in 2020 vs. CSG in 2020). And secondly, a dichotomous three‐level measure splitting CSG‐receipt on the household level, which is coded as zero for no CSG exposure in 2020, one for individuals reporting exposure to the May scale‐up, the on average more increased scale‐up, and two for individuals reporting the June scale‐up (0 = No CSG in 2020, 1 = May scale‐up, 2 = June scale‐up). Splitting the timing of CSG receipt provides us with the opportunity to understand if an increased scale‐up, that is, a marginally reduced budget constraint, is indeed more protective for individual health. As reporting of the scale‐up depends on the timing of the interview, individuals reporting the June scale‐up will have been exposed to the May scale‐up.

### 3.4 Descriptive statistics

Sample characteristics are balanced between those individuals exposed and unexposed to the lockdown‐induced income shock (Table 1). Some small variations occur in for instance self‐rated health which is on average higher among exposed prior 2020 and lower in 2020 compared to unexposed and in educational outcomes with a larger proportion in the exposed group stating that secondary education is their highest educational achievement (69% in exposed vs. 64% in unexposed group). Overall, 2319 individuals report not to live in a CSG receiving household.1042 individuals report to live in a household which is exposed to the CSG May scale‐up.2976 individuals report exposure to the CSG June scale‐up.

**TABLE 1:** Descriptive statistics by exposure status

	Unexposed (no loss of main source of household income) (*n* = 3761)	Exposed (loss of main source of household income) (*n* = 2676)
A: Outcome		
*Before*: Self‐rated health	2.87 (1.05)	2.92 (1.01)
*After*: Self‐rated health	2.19 (1.13)	2.00 (1.14)
B: Individual characteristics		
Male	0.37	0.41
Age: <15	0.08	0.06
Age: 15–24	0.23	0.21
Age: 25–34	0.24	0.29
Age: 35–44	0.18	0.23
Age: 45–54	0.11	0.12
Age: 55–64	0.08	0.06
Age: 65+	0.08	0.04
Black	0.85	0.91
Mixed‐race	0.10	0.07
Asian	0.01	0.01
White	0.04	0.02
Chronic health problem: HIV, tuberculosis, diabetes, lunge/heart condition	0.14	0.14
No education	0.06	0.05
Primary education	0.22	0.19
Secondary education	0.64	0.69
Tertiary education	0.08	0.06
C: Household characteristics		
Household members	5.46 (3.35)	5.48 (3.32)
1st Quartile of real total household assets per capita	0.25	0.25
2nd Quartile	0.25	0.25
3rd Quartile	0.24	0.26
4th Quartile	0.26	0.23
No child support grant in May or Jun 2020	0.38	0.33
Child support grant May 2020 scale‐up	0.16	0.17
Child support grant Jun 2020 scale‐up	0.46	0.50
D: COVID‐19 characteristics		
Behavioral change due to COVID‐19	0.90	0.92
Individual or anyone in household tested/screened for COVID‐19	0.36	0.40
Individual tested positive for COVID‐19	0.07	0.06
Likely to get infected with COVID‐19: Yes	0.27	0.28
Likely to get infected with COVID‐19: No	0.60	0.60
Likely to get infected with COVID‐19: Unsure	0.12	0.12
E: Geographical characteristics		
Location: Traditional	0.40	0.44
Location: Urban	0.54	0.51
Location: Farms	0.06	0.04

*Note*: Means of variables with standard deviations in parenthesis; Means for Panel B, C and E are computed over the full available data, from 2008 until 2020; means for Panel A are split into the time before and after the lockdown shock, that is, before 2020 and in 2020. Means for Panel D are computed on data in 2020. Assets are adjusted to inflation with baseline December 2016 = 100. Individual tested positive for COVID‐19 relates to those tested for COVID‐19 not the full sample. The number of individuals in the "Unexposed" group is 3761 and the number of observations over the full period is 17,812; the number of individuals in the "Exposed" group is 2676 and the number of observations over the full period is 12,676.

**FIGURE 2:**
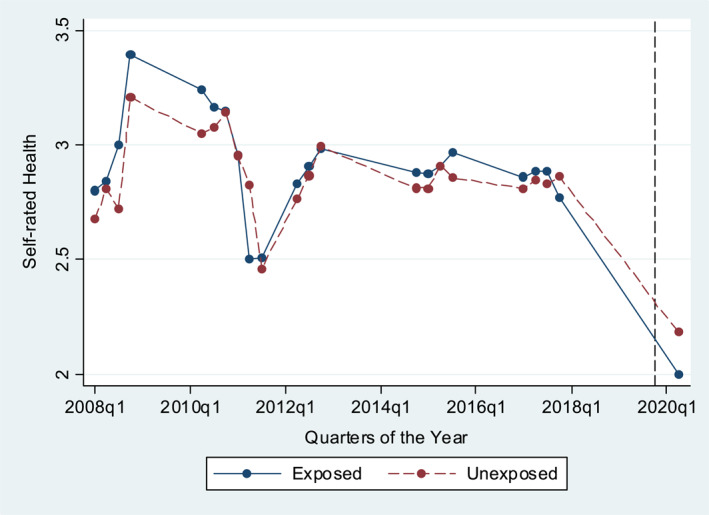
Parallel trends in health by exposure status. We present in this figure the mean values of self‐rated health in its evolution over time by exposure status and collapsed by quarters of the year. The dashed vertical line highlights the timing of the lockdown

Figure 2 provides a first visual evidence of strong parallel pre‐shock trends in unexposed and exposed groups in health. Self‐rated health follows not just similar trends in the pre‐lockdown period but also similar levels over the course of 10 years before the shock. It is also evident that individual health sees a major drop in both exposed and unexposed groups following the lockdown, with the exposed being worse off. We discuss and assess parallel trends in more detail, following the methodological discussion of the DD and heterogeneous effect DD approach.

## 4 METHODS

### 4.1 Understanding the causal effects of the income shock

#### 4.1.1 Difference‐in‐difference estimation

We use difference‐in‐difference (DD) estimation to identify the causal effects of the lockdown induced income shock on health. We estimate specification (1) for individual health as follows:

(1) $$Y_{\left(i,t\right)}=\alpha_{1}+\beta_{1}{EXPOSURE}_{\left(i,t\right)}*{POST}_{\left(i,t\right)}+\gamma_{1}{EXPOSURE}_{\left(i,t\right)}+\delta_{1}{POST}_{\left(i,t\right)}+q_{t}+\epsilon_{\left(i,t\right)}$$

Where $Y_{\left(i,t\right)}$ is health at time *t* for individual *i*, ${EXPOSURE}_{\left(i,t\right)}$ is the binary exposure indicator taking value one for exposed and zero for unexposed, ${POST}_{\left(i,t\right)}$ is the before‐and‐after dummy variable taking value one for the post‐intervention period and zero for the pre‐intervention period, and the interaction of ${EXPOSURE}_{\left(i,t\right)}*{POST}_{\left(i,t\right)}$ being the DD‐estimator with $\beta_{1}$ indicating the average causal effect of the lockdown shock on health. $q_{t}$ are quarter of year fixed effects and $\epsilon_{\left(i,t\right)}$ is the individual error term. We cluster standard errors in all estimation on the panel level, which is the individual (Cameron & Miller, 2015; Wooldridge, 2001).

### 4.2 Understanding the causal effects of the income shock by wealth quartiles

We approach the analysis of distributional effects of the income shock on health by wealth quartiles using heterogeneous effect difference‐in‐difference estimation. We estimate specification (2) as follows:

(2) $$Y_{\left(i,t\right)}=\alpha_{2}+\beta_{2}{EXPOSURE}_{\left(i,t\right)}*{POST}_{\left(i,t\right)}+\sum_{n=2}^{4}\beta_{n+1}{EXPOSURE}_{\left(i,t\right)}*{POST}_{\left(i,t\right)}*Q_{\left(i\right)}^{n}+\sum_{n=2}^{4}\beta_{n+4}{EXPOSURE}_{\left(i,t\right)}*Q_{\left(i\right)}^{n}+\sum_{n=2}^{4}\beta_{n+7}{{POST}_{\left(i,t\right)}}_{\left(i,t\right)}*Q_{\left(i\right)}^{n}+\sum_{n=2}^{4}\beta_{n+10}Q_{\left(i\right)}^{n}+\gamma_{2}{EXPOSURE}_{\left(i,t\right)}+\delta_{2}{POST}_{\left(i,t\right)}+q_{t}+\epsilon_{\left(i,t\right)}$$

Where $Q_{\left(i\right)}^{4}$ represents a dummy variable indicating the fourth quartile of real total per capita household assets define by 2017/2018 values (i.e., wealth quartile), with $Q_{\left(i\right)}^{3}$ the third quartile and $Q_{\left(i\right)}^{2}$ the second quartile. $\beta_{5}$ is the heterogeneous effect DD‐estimator for the fourth quartile of the income shock on health having netted out the effects of the first quartile heterogenous DD presented by $\beta_{2}$; $\beta_{4}$ and $\beta_{3}$ are the heterogeneous effect DD‐estimators for the third and second wealth quartile, respectively. We estimate the heterogeneous effect DD with the full interaction of dummy variables.

We further estimate specification (2) with only one heterogeneous effect DD‐term using a dummy variable pooling $Q_{\left(i\right)}^{4}$, $Q_{\left(i\right)}^{3}$, and $Q_{\left(i\right)}^{2}$ . We aim to understand with this altered specification if individuals classified as belonging to the lowest wealth quartile are disproportionately affected compared to all other individuals.

### 4.3 Understanding mitigation effects of the CSG on the lowest wealth quartile

To test our hypothesis of mitigation effects of the CSG and the scale‐up on individual health for the most vulnerable in the population, we use heterogeneous effect DD estimation with CSG‐measures on the sub‐group of individuals defined as being in the lowest wealth quartile. Using the lowest wealth quartile is motivated by identifying the economically most vulnerable populations and by the nature the of CSG which is targeted to low‐income groups. We first test the general protective effects of the CSG a specified in (3) for health:

(3) $$Y_{\left(i,t\right)}=\alpha_{3}+\beta_{15}{EXPOSURE}_{\left(i,t\right)}*{POST}_{\left(i,t\right)}+\beta_{16}{EXPOSURE}_{\left(i,t\right)}*{POST}_{\left(i,t\right)}*{CSG}_{\left(i\right)}+\beta_{17}{EXPOSURE}_{\left(i,t\right)}*{CSG}_{\left(i\right)}+\beta_{18}{POST}_{\left(i,t\right)}*{CSG}_{\left(i\right)}+\beta_{19}{CSG}_{\left(i\right)}+\gamma_{3}{EXPOSURE}_{\left(i,t\right)}+\delta_{3}{POST}_{\left(i,t\right)}+q_{t}+\epsilon_{\left(i,t\right)}$$

Where ${CSG}_{\left(i\right)}$ is a dummy variable indicating if the individual lives in a CSG‐receiving household in the post‐shock period. $\beta_{16}$ is the heterogeneous effect DD‐estimator of health effects for CSG recipients that have experienced the income shock netting out the estimated effect of the income shock on health for individuals that are not protected by the CSG in the post‐shock period, indicated by $\beta_{15}.$ We test variations in transfer amounts exploiting changes in government policies as specified in (4):

(4) $$Y_{\left(i,t\right)}=\alpha_{4}+\beta_{20}{EXPOSURE}_{\left(i,t\right)}*{POST}_{\left(i,t\right)}+\beta_{21}{EXPOSURE}_{\left(i,t\right)}*{POST}_{\left(i,t\right)}*{CSG-MAY}_{\left(i\right)}+\beta_{22}{EXPOSURE}_{\left(i,t\right)}*{POST}_{\left(i,t\right)}*{CSG-JUNE}_{\left(i\right)}+\beta_{23}{EXPOSURE}_{\left(i,t\right)}*{CSG-MAY}_{\left(i\right)}+\beta_{24}{POST}_{\left(i,t\right)}*{CSG-MAY}_{\left(i\right)}+\beta_{25}{EXPOSURE}_{\left(i,t\right)}*{CSG-JUNE}_{\left(i\right)}+\beta_{26}{POST}_{\left(i,t\right)}*{CSG-JUNE}_{\left(i\right)}+\beta_{27}{CSG-MAY}_{\left(i\right)}+\beta_{28}{CSG-JUNE}_{\left(i\right)}+\gamma_{4}{EXPOSURE}_{\left(i,t\right)}+\delta_{4}{POST}_{\left(i,t\right)}+q_{t}+\epsilon_{\left(i,t\right)}$$

Where ${CSG-MAY}_{\left(i\right)}$ is a dummy variable indicating the CSG May scale‐up and ${CSG-JUNE}_{\left(i\right)}$ a dummy variable indicating the CSG June scale‐up. Consequentially, $\beta_{21}$ is the heterogeneous effect DD‐estimator of CSG May scale‐up effects on health, $\beta_{22}$ is the heterogeneous effect DD‐estimator of the CSG June scale‐up effects on health, having netted out the effects of the income shocks on health for individuals not protected by the CSG estimated with $\beta_{20}$. Jointly estimating and thus controlling for the May and June top‐up effects permits to understand the isolated respective scale‐up effects. This is important as individuals exposed to the June scale‐up will have been exposed to earlier May scale‐up.

### 4.4 Identification assumptions

Causal inference of DD and the heterogeneous effect DD estimation relies on the assumption that the outcome variable follows the same trend in both the treatment and control group in the post‐intervention period had treatment not happened (Cunningham, 2021). Assessing parallel trends in outcomes in the period before treatment (here the shock) occurred is the common approach to test the plausibility of this assumption (Angrist & Pischke, 2008). Recent research advances in DD methods show that for the plausibility of parallel trends to hold the levels of the outcomes should ideally be similar too (Kahn‐Lang & Lang, 2020). Using Figure 2, we have shown that both conditions are satisfied.

To further assess the parallel trends, we empirically test for parallel trends in outcomes in the pre‐shock period. Doing so for the DD specifications, we regress the outcomes on the full interaction of quarters of the year with $D_{\left(i,t\right)}^{1}$ the exposure indicator for the period before the intervention occurred. For the heterogeneous effect DD specifications, we regress the outcomes on the full interaction of quarters of the year with $D_{\left(i,t\right)}^{1}$ and the specific heterogeneous effect DD‐model (i.e., quartile of wealth quartiles, CSG or CSG scale‐up levels) and test whether the effects of the specific heterogeneous effect DD‐model are jointly significantly different from zero in the pre‐shock period. No significance of the interaction effect in DD and the joint heterogeneous effect DD interaction effects is supporting evidence for the parallel trends to hold. These tests are standard practice in the literature (Angrist & Pischke, 2008; Muralidharan & Prakash, 2017). We provide findings from the parallel trend tests alongside the DD and heterogeneous effect DD‐estimations. To preface the results, we find that parallel trends hold in all models.

In the context of our study, statistical testing and graphical illustrations provide strong evidence for parallel trends in health. However, we have further arguments to support the assumption. For instance, policies or trends on the sub‐national level could have affected health and the composition of exposed and unexposed groups if exposed and unexposed had different exposure to such policies. Accordingly, we show that this is not a concern in map A1 in the supplementary material. The map illustrates South Africa divided by its 52 districts. The top picture presents the distribution of unexposed in the pre‐shock period as per district share of unexposed of the total number of unexposed. Darker shades of green imply a higher share. The same presentation is done for exposed in the picture below. Comparing the shades of the two maps, it becomes evident that both groups are similarly distributed across the country by districts. Therefore, both groups should have similar exposure to sub‐national policies before 2020.

Another important factor in determining whether it is reasonable to assume parallel trends in outcomes is the similarity of the socio‐demographic composition of the compared groups. This is likely the case for our sample as exposed and unexposed are balanced in their characteristics (Table 1). We provide further supporting evidence by illustrating pre‐trends in time‐varying demographic characteristics age, education, and household size which are all overlapping (see Figures A1, A2, and A3 in the supplementary material).

### 4.5 Robustness of findings

We assess the robustness of our findings with regards to the following five potential threats to causal inference: (1) assessing potential confounding of health due to individual characteristics including unobserved individual heterogeneity in health and COVID‐19 behaviors; (2) Confounding of health due to other health outcomes; (3) plausibility of self‐rated health as good health measure; (4) assumption of stability of wealth levels over time; and (5) assessing the sensitivity of findings to variations to pre‐treatment waves and panel composition. We present a detailed description of each robustness test in the supplementary material.

## 5 RESULTS

We present firstly findings from the DD estimation of the income shock on health, secondly findings from the heterogeneous effect DD estimation by wealth quartiles, thirdly results from the DD mitigation effect analysis of the CSG cash transfer on health for the lowest wealth quartile, and lastly findings from the robustness analysis.

### 5.1 Causal effects of the income shock on average and by wealth quartiles

Findings from the DD analysis show a strong and significant average reduction in health of 0.237 points or 0.2 standard deviations (SD) due to the income shock (Table 2, column 1). The negative health effect of the shock does not vary across the wealth distribution, as no significant difference of the effect for the second, third, and fourth wealth quartile is found when compared to the significant and negative effect of −0.256 for the first and lowest wealth quartile (Table 2, column 2). Pooling the second, third, and fourth wealth quartile together and comparing to the first wealth quartile, the effects remain unchanged (column 4). The two bottom rows of the table present findings from the parallel trend tests. Statistically, we have strong evidence for parallel trends to hold in all models as no significant differences in pre‐shock period are observed indicated by *p*‐values > 0.1. As heterogeneous effect DD estimates are presented as net effects of the first wealth quartile, we also present the total effects of each respective quartile and of the aggregated quartiles on health, computed as linear combinations. The results show that the income shock has significant negative health effects across the wealth distribution in both models (column 3 and 5).

**TABLE 2:** Difference‐in‐Difference and heterogeneous difference‐in‐difference analysis by wealth for health

	(1)	(2)	(3)	(4)	(5)
		Wealth quartiles	Linear combinations model (2)	1st wealth quartile versus rest	Linear combinations model (4)
DD	−0.237*** (0.032)				
DD 1st wealth quartile		−0.257*** (0.065)	−0.257*** (0.065)	−0.257*** (0.065)	−0.257*** (0.065)
DD 2nd wealth quartile		0.056 (0.092)	−0.202*** (0.066)		
DD 3rd wealth quartile		0.057 (0.090)	−0.200*** (0.062)		
DD 4th wealth quartile		−0.003 (0.089)	−0.260*** (0.062)		
DD 2nd+3rd+4th wealth quartile				0.028 (0.074)	−0.229*** (0.037)
Constant	2.714*** (0.030)	2.723*** (0.036)	2.723*** (0.036)	2.724*** (0.036)	2.724*** (0.036)
Observations	30,490	30,490	30,490	30,490	30,490
Individuals	6437	6437	6437	6437	6437
R‐squared	0.094	0.098	0.094	0.094	0.098
Time effects	Yes	Yes	Yes	Yes	Yes
Covariates	No	No	No	No	No
District fixed effects	No	No	No	No	No
Individual fixed effects	No	No	No	No	No
F‐Stat: Parallel trends	1.652	1.725	1.725	1.285	1.285
Prob > F: Parallel trends	0.119	0.141	0.141	0.277	0.277

*Note*: Individual clustered standard errors in parentheses; ***p < 0.01, **p < 0.05, *p < 0.1; The outcome variable is individual self‐rated health, with higher values indicating better health. We control for the fully interacted difference‐in‐difference framework but present only the difference‐in‐difference estimators for each model. DD stands for difference‐in‐difference. Findings presented in column (1) relate to Equation (1), with DD being the $\beta_{1}$coefficient. Findings in column (2) relate to Equation (2), with “DD first wealth quartile” being the $\beta_{2}$coefficient, and “DD second wealth quartile”, “DD third wealth quartile”, and “DD fourth wealth quartile” representing $\beta_{3},\;\beta_{4}\;and\;\beta_{5}$ respectively.

### 5.2 Mitigation effects of the CSG on the lowest wealth level

Findings from the mitigation effect analysis of the CSG cash transfer program on health for the first wealth quartile. Show that I those individuals who were exposed to the income shock but not protected by the CSG have a statistically significant and largest loss in health of 0.474 units or 0.4 SD in health (Table 3, columns 1). The protective effect of the CSG for individuals exposed to the income shock is significant and of size 0.3 (0.25 SD in health), meaning that individuals exposed to the shock and protected by the CSG are presenting 0.3 units better health compared to individuals exposed to the shock but unprotected by the CSG (column 1). This is a substantial effect as the cash transfer nearly fully mitigates the negative health effects from the income shock. The largest protective health effects materialize for those individuals exposed to the on average larger CSG scale‐up in May. For those individuals is the negative heath impact of the income shock fully mitigated due to the significant and substantial positive effect of 0.493 (0.5 SD in health). This is larger in magnitude than the negative health effect for non‐recipients (Table 3, column 3). In comparison, the on average lower scale‐up of the CSG in June provides no protection as the effect is not significantly different from the observed negative effect for non‐recipients. All models pass the parallel trends test as *p*‐values > 0.1.

**TABLE 3:** Heterogeneous difference‐in‐difference analysis of cash transfer mitigation effects for the lowest wealth quartile for health

	(1)	(2)	(3)	(4)
	CSG: 1st wealth quartile	Linear combinations model (1)	CSG scale‐up: 1st wealth quartile	Linear combinations model (3)
DD No CSG	−0.474*** (0.120)	−0.474*** (0.120)	−0.474*** (0.120)	−0.474*** (0.120)
DD CSG May 2020 scale‐up			0.493** (0.210)	0.019 (0.172)
DD CSG Jun 2020 scale‐up			0.240 (0.147)	−0.234*** (0.085)
DD CSG	0.301** (0.142)	−0.173** (0.077)		
Constant	2.645*** (0.072)	2.645*** (0.072)	2.645*** (0.072)	2.645*** (0.072)
Observations	7626	7626	7626	7626
Individuals	1561	1561	1561	1561
R‐squared	0.119	0.119	0.120	0.120
Time effects	Yes	Yes	Yes	Yes
Covariates	No	No	No	No
District fixed effects	No	No	No	No
Individual fixed effects	No	No	No	No
F‐Stat: Parallel trends	0.140	0.140	0.321	0.321
Prob > F: Parallel trends	0.870	0.870	0.810	0.810

*Note*: Individual clustered standard errors in parentheses; ***p < 0.01, **p < 0.05, *p < 0.1; The outcome variable is individual self‐rated health, with higher values indicating better health. We control for the fully interacted difference‐in‐difference framework but present only the difference‐in‐difference estimators for each model. DD stands for difference‐in‐difference. Findings presented in column (1) relate to Equation (3), with “DD No CSG” being the $\beta_{15}$coefficient, and “DD CSG” being the $\beta_{16}$coefficient. Findings in column (3) relate to Equation (4), with “DD No CSG” being the $\beta_{20}$coefficient, and “DD CSG May scale‐up” and “DD CSG June 2020 scale‐up” representing $\beta_{21}\;and\;\beta_{22}$ respectively.

Using linear combinations we find that the full effect of the income shock for CSG recipients, irrespective of scale‐up level, remains significant and negative (−0.173; 0.16 SD in health); however, the effect is smaller in magnitude (column 2). It is evident that the early scale‐up in May exerts full protective health effects as the total effect is positive and not statistically significant. The later scale‐up in June reduces the negative health effects by about half the size; however, it remains still statistically significant and negative with size 0.234 (0.28 SD in health) (column 4).

### 5.3 Robustness (1): Conditioning on covariates and fixed effects

Findings from the DD analysis, the heterogeneous effect DD analysis by wealth levels, and the DD CSG mitigation effect analysis are all robust to include both covariates, district fixed effects and individual fixed effects. Significance levels and direction of effects remain unchanged and parallel trends hold in all models (Tables 4 and 5).

**TABLE 4:** Robustness: Difference‐in‐Difference and heterogeneous difference‐in‐difference analysis by wealth for health with covariates and fixed effects

	(1)	(2)	(3)	(4)	(5)	(6)
		Wealth quartiles	1st wealth quartile versus rest		Wealth quartiles	1st wealth quartile versus rest
DD	−0.225*** (0.032)			−0.218*** (0.036)		
DD 1st wealth quartile		−0.264*** (0.064)	−0.264*** (0.064)		−0.244*** (0.072)	−0.244*** (0.072)
DD 2nd wealth quartile		0.078 (0.092)			0.052 (0.103)	
DD 3rd wealth quartile		0.069 (0.089)			0.074 (0.100)	
DD 4th wealth quartile		0.031 (0.089)			−0.004 (0.100)	
DD 2nd+3rd+4th wealth quartile			0.054 (0.074)			0.036 (0.083)
Constant	2.743*** (0.097)	2.722*** (0.098)	2.739*** (0.098)	2.897*** (0.233)	2.843*** (0.237)	2.866*** (0.236)
Observations	30,490	30,490	30,490	30,490	30,490	30,490
Individuals	6437	6437	6437	6437	6437	6437
R‐squared	0.193	0.196	0.194	0.415	0.416	0.415
Time effects	Yes	Yes	Yes	Yes	Yes	Yes
Covariates	Yes	Yes	Yes	Yes	Yes	Yes
District fixed effects	Yes	Yes	Yes	Yes	Yes	Yes
Individual fixed effects	No	No	No	Yes	Yes	Yes
F‐Stat: Parallel trends	0.704	1.129	0.554	1.240	1.566	0.805
Prob > F: Parallel trends	0.402	0.341	0.575	0.266	0.181	0.447

*Note*: Individual clustered standard errors in parentheses; ***p < 0.01, **p < 0.05, *p < 0.1; The outcome variable is individual self‐rated health, with higher values indicating better health. Columns (4) to (6) include additional individual fixed effects. We control for the fully interacted difference‐in‐difference framework but present only the difference‐in‐difference estimators for each model. DD stands for difference‐in‐difference.

**TABLE 5:** Robustness: Heterogeneous difference‐in‐difference analysis of cash transfer mitigation effects for the lowest wealth quartile for health

	(1)	(2)	(3)	(4)
	CSG: 1st wealth quartile	CSG scale‐up: 1st wealth quartile	CSG: 1st wealth quartile	CSG scale‐up: 1st wealth quartile
DD No CSG	−0.483*** (0.120)	−0.485*** (0.120)	−0.453*** (0.131)	−0.453*** (0.131)
DD CSG May 2020 scale‐up		0.485** (0.209)		0.427* (0.234)
DD CSG Jun 2020 scale‐up		0.254* (0.147)		0.256 (0.161)
DD CSG	0.308** (0.142)		0.298* (0.157)	
Constant	2.520*** (0.188)	2.512*** (0.187)	2.376*** (0.363)	2.370*** (0.362)
Observations	7626	7626	7626	7626
Individuals	1561	1561	1561	1561
R‐squared	0.215	0.217	0.424	0.425
Time effects	Yes	Yes	Yes	Yes
Covariates	Yes	Yes	Yes	Yes
District fixed effects	Yes	Yes	Yes	Yes
Individual fixed effects	No	No	Yes	Yes
F‐Stat: Parallel trends	0.168	0.241	0.220	0.170
Prob > F: Parallel trends	0.845	0.868	0.803	0.917

*Note*: Individual clustered standard errors in parentheses; ***p < 0.01, **p < 0.05, *p < 0.1; The outcome variable is individual self‐rated health, with higher values indicating better health. Column (3) and (4) include additional individual fixed effects. We control for the fully interacted difference‐in‐difference framework but present only the difference‐in‐difference estimators for each model. DD stands for difference‐in‐difference.

### 5.4 Robustness (2): Conditioning on health outcomes

Conditioning the DD analysis, the heterogeneous effect DD by wealth quartiles, and the DD CSG mitigation analysis on individual chronic health problems and COVID‐19 status, results remain robust as coefficient size, significance, and direction of the effect remain unchanged (Tables A2 and A3 in the supplementary material). Parallel trends hold in all models.

### 5.5 Robustness (3): Self‐rated health, high blood pressure and depression

Graphical illustrations of the objective health measures CES‐D and high blood pressure indicate parallel trends and similar levels between exposed and unexposed groups over a 10‐year period prior to the occurrence of the shock (Figures A4 and A5 in the supplementary material). These findings are corroborated in the statistical analysis (Table A4 columns (1) and (2) in the supplementary material).

We further find a strong significant and negative association of both variables with self‐rated health which is evidence of the construct validity of our main outcome measure self‐rated health (Table A5 in the supplementary material). CES‐D and high blood pressure exert a similar average effect on self‐rated health when comparing their standardized beta‐coefficients (column (2)), further evidencing that self‐rated health is a good general health measure among the study population. Controlling for age group and gender of the individual, CES‐D and high blood pressure retain their strong statistically significant negative relationship with self‐rated health (column (3)). The standardized beta‐coefficient (column (4)) of high blood pressure is reduced whilst the CES‐D beta coefficient remains unchanged. The change in magnitude likely occurs due to the higher prevalence of high‐blood pressure among the older population (Peltzer & Phaswana‐Mafuya, 2013).

### 5.6 Robustness (4): Stability of (low) wealth over time

Findings from the Wilcoxon signed‐rank tests show that no significant rank effects are present (Table A6 in the supplementary material), neither for the lowest per capita household income quartile across the entire NIDS‐surveys, spanning a period of 12 years, nor for the lowest per capita total household assets quartile, comparing NIDS wave five to NIDS wave four and three. Estimating the DD and heterogeneous effect DD analyses using wealth quartiles expressed in 2014/2015 values (Table A7 in the supplementary material), we observe the same pattern of effects (i.e., coefficient magnitude, significance of effects, parallel trends) as in the main specification using wealth quartiles with 2017/2018 values. This indicates, together with the results from the Wilcoxon signed‐rank tests, that our findings are robust to the timing of the choice of wealth variables and that we can indeed assume temporal stability in wealth levels across time.

### 5.7 Robustness (5): Panel composition and length of study period

Lastly, estimating the DD and heterogeneous DD analyses by wealth and by CSG‐receipt respectively, and hence using a shorter pre‐treatment time‐period from 2014 onwards, our main findings remain unchanged (see Table A8 and A9 in the supplementary material). This shows that our analysis is robust to alternative compositions of the panel and to using shorter time‐periods.

## 6 DISCUSSION

We have aimed to identify the effect of the COVID‐19 lockdown induced income shock on health among the South African population. We used longitudinal data on 6437 individuals observed across fives waves of the NIDS and the first wave of the NIDS Coronavirus Rapid Mobile Survey. Employing difference‐in‐difference analysis, we found significant evidence for on average strong depreciations in individual health equivalent to 0.2 standard deviations in health. Moving beyond mean effects, we assessed heterogeneous effects of the income shock across wealth quartiles using heterogeneous effect difference‐in‐difference analysis. We found neither a difference of the income shock on health outcomes across wealth quartiles, nor for the lowest wealth quartile. This evidence differs to our hypothesis of distributional negative health effects which was that the income shock would have more severe negative health effects on the lower income group.

Our last objective was to understand mitigation effects of scaled‐up cash transfers for the lowest wealth quartile, which provides a tenable answer for these unexpected findings. We found, as initially hypothesized, protective effects of the CSG on health. General exposure to the CSG mitigated the negative health effect by more than half the size. Further analysis by CSG scale‐up levels showed that the on average larger scale‐up of the CSG in May fully mitigated the negative health consequences of the income shock. In contrast, the on average lower scale‐up from June onwards did not fully mitigate the negative health effects; however, it still exerted protective health effects as it reduced the burden of the shock by half the size compared to individuals exposed to the shock but unprotected by the CSG. These findings can explain why we have neither seen distributional effects nor a disadvantage in health outcomes, specifically among those in the lowest wealth quartile. The rapid response to an unprecedented crisis in form of scaled‐up social safety nets for those at the bottom of the wealth distribution is a reasonable explanation for avoided inequity in health outcomes in the short run.

Causal inference of difference‐in‐difference analysis relies on the assumption that the outcome follows the same trend in the treated and untreated (exposed and unexposed to the shock) group post‐intervention had the treatment not happened (Cunningham, 2021). Commonly, parallel trends in outcomes in the pre‐treatment period (pre‐shock) between treated and untreated are tested for the plausibility of this assumption (Angrist & Pischke, 2008). We conducted various graphical and statistical analyses which all identified strong parallel trends in outcomes. We further made a compelling argument for parallel trends by showing that exposed and unexposed share not just similar trends in demographic characteristics and objective measures of health outcomes, but also similar exposure of local variations on the district level. We also showed that the findings are robust to controlling for possible confounders, COVID‐19 related outcomes such as infection risk or behavioral change, health outcomes such as chronic health conditions or a positive COVID‐19 test, and individual and district fixed effects. This evidence indicates that the (heterogeneous effect) difference‐in‐difference method successfully isolates the effect of the shock on health.

Our findings corroborate existing analysis of the effects of recession related job‐loss on health outcomes. Similar to previous studies, we found significant negative health effects of the lockdown induced income shock (loss of main source of household income) and (full) mitigation of the negative effects through social support and security programmes such as cash transfers (Hone et al., 2019; Margerison‐Zilko et al., 2016). Different to existing analyses is the context of COVID‐19 and its extreme disruption to individual livelihoods, moving far beyond the experience of a recession‐related job‐loss. A limitation of related existing research in LMIC‐settings is the focus on purely employment effects, which likely produces an underestimation of the health consequences of economic shocks (Hone et al., 2019; Margerison‐Zilko et al., 2016). This is due the nature of labor markets in LMICs, which have a large share of individuals engaged in the informal labor market rather than in formal employment (Medina et al., 2017; World Bank, 2020). Consequentially, a measure of unemployment is less likely to capture effects for populations with a larger share of informal workers, whereas a broader measure such as ours (exposure to loss of the main source of household income) reflects such variations.

Our analysis is also unique for its complexity by moving beyond mean effects and understanding distributional effects across wealth quartiles. Another important contribution is the analysis of different scale‐ups of a nation‐wide cash transfer program as an immediate response to the crisis by a national government. Our study is to our knowledge the first study to show that despite the extreme event of a nation‐wide lockdown, social protection programmes can fully mitigate the negative health if scaled‐up and implemented swiftly.

We are aware that our study has limitations. We measured wealth quartiles with financial values of household assets in 2017/2018. Financial assets will likely change to the better or worse within 2 years. However, as we use a distributional measure rather than an absolute measure of wealth, marginal changes are less relevant if the composition and distribution of assets across the population are relatively constant across time, and South Africa has indeed a low social‐mobility (World Economic Forum, 2020). We also showed that our findings are robust to using financial values of households assets in 2014/2015 and that Wilcoxon signed‐rank tests on income and wealth quartiles rejected significant changes of ranking for individuals in the lowest quartiles over time. Both finding support the argument of a stable distribution of assets across time and the appropriateness of our measure.

The scale‐ups of the CSG cash transfer program occurred successively, consequently individuals exposed to the scale‐up in June are also likely to have been exposed to the May scale‐up. This may affect the ability to isolate the true scale‐up effect in June, if parallel trends in May and June groups had been different (the effect would not be as if random), and, if we had not controlled for both May and June scale‐up effects in the estimation. However, there is no concern as we controlled in all estimations for both effects and parallel trends were observed in both groups, implying that changes in outcomes are as if random. Any change in May would have been observed for those individuals in June had they been observed in May, and vice versa. Consequentially, we identified the causal marginal change of May and June scale‐ups using heterogeneous effect difference‐in‐difference analysis.

A final limitation is the use of a subjective self‐rated health measure. It may raise the question which underlying health concept and according changes in health we observed. Self‐rated health is widely regarded as a general health measure which can pick up variations in both physical and mental health (Contoyannis & Jones, 2004). Using available data on objective physical and mental health measures in the NIDS, to test the construct validity of our self‐rated health outcome, we found that our measure indeed shows strong associations with both physical and mental health in the pre‐shock period. We also showed the presence of parallel trends in objective mental health and physical health measures along the observed parallel trends in self‐rated health in exposed and unexposed groups. Therefore, we are confident that self‐rated health is indeed picking up variations in general health which is conceptually a combination of both physical and mental health.

Our research makes important contributions to health policy Our analysis showed that nationwide lockdowns to control the spread of COVID‐19 had a significant negative impact on self‐reported health of people across all income quartiles in South Africa. However, we found that social protection programmes like cash transfer programmes, if scaled‐up, can be a powerful tool. In the South African context, they (fully) mitigated these adverse health effects for the most economically vulnerable population. Increasing cash transfer programmes at the intensive margin caused protective income effects which in turn led to protective health effects. Looking beyond COVID‐19, policy makers may consider the positive health effects alongside the economic benefits when scaling‐up social protection mechanisms in the immediate aftermath of an extreme income shock, which could result from future economic recessions, natural disasters, or other epidemics/pandemics.

Whilst our research identified the immediate effects of the income shock on health and related mitigation of cash transfer programmes in the South African context, future research should identify the mid‐to long‐term consequences and mitigation potential alongside the costs and benefits of such interventions, ideally comparing to alternative social protection interventions. Furthermore, ongoing research should also assess the relevance of our findings in other settings, such as lower income countries with more severe levels of economic poverty and consumer markets even more exposed to global shocks.

## CONFLICT OF INTEREST

The author has no conflict of interest to declare.

## ETHICS STATEMENT

Ethics approval is not required for this study. All data used in this study is available publicly from https://www.datafirst.uct.ac.za. As a result, no data was collected directly from human subjects.

## Supporting information

Supplementary Material

## Data Availability

The data that support the findings of this study are openly available at https://www.datafirst.uct.ac.za/dataportal/index.php/catalog.

## References

[hec4592-bib-0001] Angrist, J. D. , & Pischke, J.‐S. (2008). Mostly harmless econometrics: An empiricist’s companion. Princeton University Press.

[hec4592-bib-0002] Arndt, C. , Davies, R. , Gabriel, S. , Harris, L. , Makrelov, K. , Robinson, S. , Levy, S. , Simbanegavi, W. , van Seventer, D. , & Anderson, L. (2020). Covid‐19 lockdowns, income distribution, and food security: An analysis for South Africa. Global Food Security, 26, 100410. 10.1016/j.gfs.2020.100410 32834955 PMC7366977

[hec4592-bib-0003] Beck, S. , Sebastián, M. S. , Beck, S. , & Sebastián, M. S. (2015). Basic income – healthy outcome? Effects on health of an Indian basic income pilot project: A cluster randomised trial. Journal of Development Effectiveness, 7(1), 111–126. Routledge. 10.1080/19439342.2014.974200

[hec4592-bib-0004] Benhura, M. , & Magejo, P. (2021). National income Dynamics study (NIDS) ‐ Coronavirus rapid mobile survey (CRAM): Who cannot work from home in South Africa? Evidence from wave 4 of NIDS‐CRAM.

[hec4592-bib-0005] Bilal, U. , Cooper, R. , Abreu, F. , Nau, C. , Franco, M. , & Glass, T. A. (2017). Economic growth and mortality: Do social protection policies matter? International Journal of Epidemiology, 46(4), 1147–1156. 10.1093/ije/dyx016 28338775

[hec4592-bib-0006] Burger, R. , Posel, D. , & von Fintel, M. (2017). The relationship between negative household events an depressive symptoms: Evidence from South African longitudinal data. Journal of Affective Disorders, 218, 170–175. 10.1016/j.jad.2017.04.031 28477493

[hec4592-bib-0007] Cameron, A. C. , & Miller, D. L. (2015). A practitioner’s guide to cluster‐robust inference. Journal of Human Resources, 50(2), 317–372. 10.3368/jhr.50.2.317

[hec4592-bib-0008] Cluver, L. , Boyes, M. , Orkin, M. , Pantelic, M. , Molwena, T. , & Sherr, L. (2013). Child‐focused state cash transfers and adolescent risk of HIV infection in South Africa: A propensity‐score‐matched case‐control study. The Lancet Global Health, 1(6), 362–370. 10.1016/s2214-109x(13)70115-3 25104601

[hec4592-bib-0009] Contoyannis, P. , & Jones, A. M. (2004). Socio‐economic status, health and lifestyle. Journal of Health Economics, 23(5), 965–995. 10.1016/j.jhealeco.2004.02.001 15353189

[hec4592-bib-0010] Cunningham, S. (2021). Causal inference: The mixtape (1st ed.). Yale Univesity Press.

[hec4592-bib-0011] Daniels, R. C. , & Khan, S. (2019). Household balance sheets in South Africa. NIDS Discussion Paper.

[hec4592-bib-0012] Delaney, A. , Ismail, Z. , Graham, L. , & Ramkissoon, Y. (2008). Review of the child support grant: Uses, implementation and obstacles. United Nations Children’s Fund Report.

[hec4592-bib-0013] Department of Health South Africa . (2021). COVID‐19 risk adjusted strategy. [Online] Retrieved from https://sacoronavirus.co.za/covid‐19‐risk‐adjusted‐strategy/

[hec4592-bib-0014] Drain, P. K. , & Garrett, N. (2020). SARS‐CoV‐2 pandemic expanding in sub‐Saharan Africa: Considerations for COVID‐19 in people living with HIV. EClinicalMedicine, 22, 100342. Elsevier Ltd. 32322805 10.1016/j.eclinm.2020.100342 PMC7174187

[hec4592-bib-0015] Dudel, C. , Garbuszus, J. M. , & Schmied, J. (2021). Assessing differences in household needs: A comparison of approaches for the estimation of equivalence scales using German expenditure data. Empirical Economics, 60(4), 1629–1659. 10.1007/s00181-020-01822-6

[hec4592-bib-0016] Fiszbein, A. , & Schady, N. (2009). Conditional cash transfers ‐ reducing present and future poverty. World Bank Policy Research Report.

[hec4592-bib-0017] Gaarder, M. M. , Glassman, A. , & Todd, J. E. (2010). Conditional cash transfers and health: Unpacking the causal chain. Journal of Development Effectiveness, 2(1), 6–50. 10.1080/19439341003646188

[hec4592-bib-0018] Gomersall, J. (2013). The performance of the child support grant: Review and research priorities. Development Southern Africa, 30(4–05), 525–544. 10.1080/0376835x.2013.830240

[hec4592-bib-0019] Grossman, M. (1972). Concept of health capital and demand for health. Journal of Political Economy, 80(2), 223–225. 10.1086/259880

[hec4592-bib-0020] Gundersen, C. , & Ziliak, J. P. (2015). Food insecurity and health outcomes. Health Affairs, 34(11), 1830–1839. 10.1377/hlthaff.2015.0645 26526240

[hec4592-bib-0021] Haider, N. , Osman, A. Y. , Gadzekpo, A. , Akipede, G. O. , Asogun, D. , Ansumana, R. , Lessells, R. J. , Khan, P. , Hamid, M. M. A. , Yeboah‐Manu, D. , Mboera, L. , Shayo, E. H. , Mmbaga, B. T. , Urassa, M. , Musoke, D. , Kapata, N. , Ferrand, R. A. , Kapata, P.‐C. , Stigler, F. , … McCoy, D. (2020). Lockdown measures in response to COVID‐19 in nine sub‐Saharan African countries. BMJ Global Health, 5(10), e003319. 10.1136/bmjgh-2020-003319 PMC7542624 33028699

[hec4592-bib-0022] Hone, T. , Mirelman, A. J. , Rasella, D. , Paes‐Sousa, R. , Barreto, M. L. , Rocha, R. , & Millett, C. (2019). Effect of economic recession and impact of health and social protection expenditures on adult mortality: A longitudinal analysis of 5565 Brazilian municipalities. The Lancet Global Health, 7(11), e1575–e1583. Elsevier. 10.1016/s2214-109x(19)30409-7 31607469

[hec4592-bib-0023] Huijts, T. , Reeves, A. , Mckee, M. , & Stuckler, D. (2015). The impacts of job loss and job recovery on self‐rated health: Testing the mediating role of financial strain and income. European Journal of Public Health, 25(5), 801–806. 10.1093/eurpub/ckv108 26045524

[hec4592-bib-0062] ILO‐OECD . (2020). The impact of the COVID‐19 pandemic on jobs and incomes in G20economies.

[hec4592-bib-0024] Ingle, K. , Brophy, T. , & Daniels, R. (2020). National income Dynamics study (NIDS) – Coronavirus rapid mobile survey (CRAM) ‐panel user manual.

[hec4592-bib-0025] Jain, R. , Budlender, J. , Zizzamia, R. , & Bassier, I. (2020). National income Dynamics study (NIDS) – Coronavirus rapid mobile survey (CRAM) ‐ the labor market and poverty impacts of Covid‐19 in South Africa.

[hec4592-bib-0026] Jebena, M. G. , Lindstrom, D. , Lachat, C. , Belachew, T. , & Kolsteren, P. (2017). The effect of food insecurity on health status of adolescents in Ethiopia: Longitudinal study. BMC Public Health, 17(1), 465. 10.1186/s12889-017-4406-5 28521757 PMC5437384

[hec4592-bib-0027] Kahn‐Lang, A. , & Lang, K. (2020). The promise and pitfalls of differences‐in‐differences: Reflections on 16 and pregnant and other applications. Journal of Business & Economic Statistics, 38(3), 613–620. Taylor & Francis. 10.1080/07350015.2018.1546591

[hec4592-bib-0028] Kerr, A. , Ardington, C. , & Burger, R. (2020). National income Dynamics study (NIDS) – Coronavirus rapid mobile survey (CRAM) ‐sample design and weighting in the NIDS‐CRAM survey.

[hec4592-bib-0029] Kim, Y. (2015). The dynamics of health and its determinants among the elderly in developing countries. Economics & Human Biology, 19, 1–12. Elsevier B.V. 10.1016/j.ehb.2015.06.001 26185895

[hec4592-bib-0030] Köhler, T. , & Bhorat, H. (2020). National income Dynamics study (NIDS) ‐ Coronavirus rapid mobile survey (CRAM): Social assistance during South Africa’s national lockdown: Examining the COVID‐19 grant, changes to the child support grant, and post‐october policy options.

[hec4592-bib-0031] Leibbrandt, M. , Woolard, I. , & De Villiers, L. (2009). Methodology: Report on NIDS wave 1. Southern African Labour & Development Research Unit.

[hec4592-bib-0032] Margerison‐Zilko, C. , Goldman‐Mellor, S. , Falconi, A. , & Downing, J. (2016). Health impacts of the great recession: A critical review. Current Epidemiology Reports, 3(1), 81–91. 10.1007/s40471-016-0068-6 27239427 PMC4880023

[hec4592-bib-0033] Mbunge, E. (2020). Effects of COVID‐19 in South African health system and society: An explanatory study. Diabetes & Metabolic Syndrome: Clinical Research & Reviews, 14(6), 1809–1814. 10.1016/j.dsx.2020.09.016 PMC7485444 32956925

[hec4592-bib-0034] Medina, L. , Jonelis, A. , & Cangul, M. (2017). The informal economy in sub‐saharan Africa: Size and determinants. (IMF Working Paper).

[hec4592-bib-0035] Muralidharan, K. , & Prakash, N. (2017). Cycling to School: Increasing secondary School enrollment for girls in India. American Economic Journal: Applied Economics, 9(3), 321–350. 10.1257/app.20160004

[hec4592-bib-0036] Mwabu, G. (2007). Chapter 53 health economics for low‐income countries. Handbook of Development Economics, 4(07), 3305–3374. 10.1016/s1573-4471(07)04053-3

[hec4592-bib-0037] O’Donnell, O. , van Doorslaer, E. , Wagstaff, A. , & Lindelow, M. (2008). Analyzing health equity using household survey data. The World Bank.

[hec4592-bib-0038] OECD . (2011). Divided we stand. Why Inequality Keeps Rising.

[hec4592-bib-0039] Pega, F. , Liu, Y. S. , Walter, S. , Lhachimi, S. K. , & Saith, R. (2017). Unconditional cash transfers for reducing poverty and vulnerabilities: Effect on use of health services and health outcomes in low‐ andmiddle‐income countries. Cochrane Database of Systematic Reviews, 11. 10.1002/14651858.cd011135 PMC6486161 29139110

[hec4592-bib-0040] Peltzer, K. , & Phaswana‐Mafuya, N. (2013). Hypertension and associated factors in older adults in South Africa. Cardiovascular Journal of Africa, 24(3), 67–71. Clinics Cardive Publishing. 23736129 10.5830/CVJA-2013-002 PMC3721893

[hec4592-bib-0041] Poletti, P. , Tirani, M. , Cereda, D. , Trentini, F. , Guzzetta, G. , Sabatino, G. , Marziano, V. , Castrofino, A. , Grosso, F. , Del Castillo, G. , Piccarreta, R. , Andreassi, A. , Melegaro, A. , Gramegna, M. , Ajelli, M. , Merler, S. , & ATS Lombardy COVID‐19 Task Force . (2021). Association of age with likelihood of developing symptoms and critical disease among close contacts exposed to patients with confirmed SARS‐CoV‐2 infection in Italy. JAMA Network Open, 4(3), e211085–e211085. 33688964 10.1001/jamanetworkopen.2021.1085 PMC7948061

[hec4592-bib-0042] Porreca, E. , & Rosati, F. C. (2019). The impact of cash transfer programmes on youth and adult labour supply: Evidence from Lesotho and the Philippines. Journal of International Development, 31(4), 291–311. John Wiley & Sons, Ltd. 10.1002/jid.3405

[hec4592-bib-0043] Salyer, S. J. , Maeda, J. , Sembuche, S. , Kebede, Y. , Tshangela, A. , Moussif, M. , Ihekweazu, C. , Mayet, N. , Abate, E. , Ouma, A. O. , & Nkengasong, J. (2021). The first and second waves of the COVID‐19 pandemic in Africa: A cross‐sectional study. The Lancet, 397(10281), 1265–1275. Elsevier. 10.1016/s0140-6736(21)00632-2 PMC8046510 33773118

[hec4592-bib-0044] Schiele, V. , & Schmitz, H. (2016). Quantile treatment effects of job loss on health. Journal of Health Economics, 49, 59–69. Elsevier B.V. 10.1016/j.jhealeco.2016.06.005 27376909

[hec4592-bib-0045] Skoufias, E. , & Di Maro, V. (2008). Conditional cash transfers, adult work incentives, and poverty. Journal of Development Studies, 44(7), 935–960. 10.1080/00220380802150730

[hec4592-bib-0046] Skoufias, E. , Unar, M. , & Gonzalez de Cossio, T. (2013). The poverty impacts of cash and in‐kind transfers: Experimental evidence from rural Mexico. Journal of Development Effectiveness, 5(November), 401–429. 10.1080/19439342.2013.843578

[hec4592-bib-0047] South African Government (2021). About (COVID‐19) alert system. [Online] Retrieved from https://www.gov.za/covid‐19/about/about‐alert‐system

[hec4592-bib-0048] Statistics South Africa (SSA) (2020a). Gross domestic product ‐ first quarter 2020.

[hec4592-bib-0049] Statistics South Africa (SSA) (2020b). Quarterly labour force survey ‐ quarter (Vol. 1).2020.

[hec4592-bib-0050] Statistics South Africa (SSA) (2021). Quarterly labour froce survey. Quarter 2: 2021.

[hec4592-bib-0051] Strauss, J. , & Thomas, D. (1998). Health, nutrition, and economic development. Journal of Economic Literature, 36(2), 766–817.

[hec4592-bib-0052] UNCTAD . (2020). COVID‐19: A threat to food security in Africa. [Online] Retrieved from https://unctad.org/en/pages/newsdetails.aspx?OriginalVersionID=2450

[hec4592-bib-0053] UNICEF, DSD and SASSA (2012). The South African child support grant impact assessment: Evidence from a survey of children, adolescents and their households. (United Nations Children’s Fund, Department of Social Development South Africa and South African Social Security Agency Report).

[hec4592-bib-0054] Wagstaff, A. (1986). The demand for health: Some new empirical evidence. Journal of Health Economics, 5(3), 195–233. 10.1016/0167-6296(86)90015-9 10279033

[hec4592-bib-0055] WHO (2020). COVID‐19 could deepen food insecurity, malnutrition in Africa. [Online] Retrieved from https://www.afro.who.int/news/covid‐19‐could‐deepen‐food‐insecurity‐malnutrition‐africa

[hec4592-bib-0056] Wills, G. , Patel, L. , van der Berg, S. , & Mpeta, B. (2020). National Income Dynamics Study (NIDS) – Coronavirus Rapid Mobile Survey (CRAM) ‐ household resource flows and food poverty during South Africa’s lockdown: Short‐term policy implications for three channels of social protection.

[hec4592-bib-0057] Wittenberg, M. , & Leibbrandt, M. (2017). Measuring inequality by asset indices: A general approach with application to South Africa. Review of Income and Wealth.

[hec4592-bib-0058] Wooldridge, J. M. (2001). Econometric analysis of cross section and panel data. The MIT Press.

[hec4592-bib-0059] World Bank . (2020). The World Bank data: Informal employment. [Online] Retrieved from https://data.worldbank.org/indicator/SL.ISV.IFRM.ZS

[hec4592-bib-0060] World Economic Forum . (2020). The global social mobility report 2020 equality. Opportunity and a New Economic Imperative.

