# Supplementary material for: Economic shocks, health, and social protection: The effect of COVID‐19 income shocks on health and mitigation through cash transfers in South Africa
Source: Health Econ. 2022 Aug 23;31(11):2481–98. doi: 10.1002/hec.4592 (PMC9539133; doi:10.1002/hec.4592)
Supplement: Supplementary file 1 — Supplementary Material [file HEC-31-2481-s001.docx]

*APPENDIX: Economic shocks, health, and social protection: The effect of COVID-19 income shocks on health and mitigation through cash transfers in South Africa*

*Robustness tests*

*Robustness (1): Conditioning on covariates and fixed effects*

We re-estimate specifications (1) to (4) and the parallel trends tests, by firstly controlling for a set of covariates and district fixed effects identified as potential confounders of the health and income shock relationship (Strauss and Thomas, 1998; Deaton, 2003; Ohrnberger et al., 2020). These covariates are gender and race (black, mixed-race, white, other), the age-group of the individual (<15, 15-24, 25-34, 35-44, 45-54, 55-64,65+), highest educational achievement (no education, primary, secondary, or tertiary education), the number of household members including children, and the location of the household (rural, urban, farms). The intuition of controlling for district fixed effects is that for instance health care provision or the labour market may differ on such levels, which could potentially affect the DD and heterogeneous effect DD estimators.

We are also controlling for COVID-19 behaviours. These covariates are a set of dummy variables indicating individual perceived risk of infection (no risk, at risk, unsure), a binary variable indicating if the individual or a household member was tested for COVID-19 which we use as proxy for household level risk factor of COVID-19, and another binary variable indicating if the individual has changed his/her behaviour due to COVID-19. The motivation of controlling for these variables is that the individual scoring of self-rated health may depend on variations in COVID risk factors. However, we do not expect significant changes in the magnitude of DD and heterogeneous effect DD effects as the descriptive statistics have shown good balance in these characteristics. Further indicate the parallel and overlapping trends in self-rated health between exposed and unexposed across 10 years prior 2020 that individuals in both groups evaluate their health similarly across time and different events such as in the aftermath of the global financial crisis in the years after 2008 or over the course of the HIV-epidemic in South Africa.

Secondly, we control for the effect of unobserved individual heterogeneity adding individual fixed effects to the estimation. The intuition for individual fixed effects is that unobserved constant idiosyncratic factors such as health investment preferences may affect health outcomes and bias estimates. We conduct Hausman tests which all reject random effects in favour of fixed effects. If DD and heterogeneous effect DD estimation correctly identify the effects of the income shock on health, adding covariates and controlling for various fixed effects should not drastically alter the magnitude of the effects. Otherwise, there may be concern that other unobserved factors further effect the estimates.

*Robustness (2): Conditioning on health outcomes*

Self-rated health may be influenced by individual health outcomes. As we observe strong parallel trends in self-rated health in the pre-shock period over a time of ten years, we believe that such bias is unlikely to occur. Further show the descriptive statistics good balance in health characteristics between exposed and unexposed. Nevertheless, we re-estimate all models using the above-mentioned set of covariates and the following two additional variables. Firstly, a binary variable indicating if the individual has reported a chronic health problem (HIV, TB, diabetes, lunge condition or heart condition). Using chronic health problems, we can control for variations in health profiles of exposed and unexposed groups over time. Secondly, a binary variable indicating if the individual reported to have had a positive COVID-19 test result. The variable takes value zero for all other individuals, including those that did not have a COVID-19 test. Using self-reported test results, we can control for variations in COVID-19 health effects that could potentially bias the DD and heterogeneous effect DD estimator of the shock on self-rated health.

*Robustness (3): Self-rated health, high blood pressure and depression*

A criticism of self-rated health is the subjective nature of the measure. Arguably, this is of limited concern in our study as we are observing self-rated health across time for the same individuals and are differencing out the absolute value of the measure using DD and heterogeneous effect DD. Consequentially, we are using relative individual variations in self-rated health rather than absolute. However, we still provide an assessment of the plausibility of self-rated health as good general health measure in the context of our study populations. We observe two objective health measures in the five waves of the NIDS pre-dating the time of the shock. The first is an indicator of high blood pressure, computed as a binary variable over two measurements of systolic and diastolic blood pressure which is measured at the time of the interview. Individuals have high blood pressure if either their systolic value is equal or greater than 140 or their diastolic measure is equal or greater than 90 (Mayo Clinic, 2020). High blood pressure is covering the physical health dimension.

The second measure is the Centre for Epidemiological Depression Scale 10-item version (CES-D) widely regarded as strong measure for depression across populations and time which has also been validated for the South African population (Radloff, 1977; Armenta et al., 2014; Baron et al., 2017). The CES-D is computed over 10 items related to positive and negative emotional effect such as happiness, loneliness, sleep deprivation and depressive feelings. For each of the 10-items the respondents reported how frequently they have experienced symptoms in the past week, from “not at all (less than one day)” to “all of the time (five to seven days)”. The CES-D ranges from 0-30 with higher values indicating worse mental health (worse depressive symptoms).

We firstly assess parallel trends in high blood pressure and CES-D for exposed and unexposed using graphical illustrations and the previously described statistical parallel trends tests. Parallel trends in both measures would provide more evidence that differences in health are not driven by differences in health prior to the shock. Also, since we observe parallel trends in self-rated health, passed parallel trends for CES-D and high blood pressure would add support the usefulness of this measure. Secondly, we check if self-rated health indeed is a good general health measure by regressing self-rated health on CES-D and high blood pressure in the pre-shock period controlling for time trends. As higher values in both high blood pressure and CES-D imply worse health, we would expect a negative association of these measures with the positively coded self-rated health.

*Robustness (4): Stability of (low) wealth over time*

We assume that individuals belonging to the lowest wealth quartile in 2017/2018 are likely to have remained in this position until early 2020. Whilst empirical evidence supports our assumption of low social mobility in South Africa (World Economic Forum, 2020), we also test the assumption in two ways.

Firstly, we present Wilcoxon signed-rank tests on temporal variations in ranks of income and wealth quartiles. We conduct these tests by firstly comparing the lowest quartile of real per capita household income to all others. We add income as it is observed in all waves. The computation of real per capita household follows the approach used to compute real total per capita assets. Secondly, we compute the Wilcoxon signed-rank test comparing the lowest quartiles of real per capita total to the rest. Household assets are observed in waves two, four and five of the NIDS. The null hypothesis of Wilcoxon signed-rank tests is no difference in the distribution, consequentially failing to reject the hypothesis will indicate stability in maintaining lowest income and wealth levels over time. Secondly, we re-estimate models (1) to (4) using total asset quartiles defined in 2014/15 values (wave four of NIDS). We expect similar results if our argument of low mobility over time is to hold.

*Robustness (5): Panel composition and length of study period*

We lastly assess the sensitivity of our analysis to the length and composition of our unbalanced panel. Doing so, we re-estimate the analysis presented in table (2), the DD and heterogenous DD effects by wealth quartiles, and the analysis presented in table (3), DD heterogeneity by CSG exposure, for a shorter pre-intervention period, using the panel from 2014 onwards which is half of the initial time-period. Thereby, we drop observations from preceding waves (i.e. observations in 2008, 2010/11, 2012). This approach also helps understanding if the findings are sensitive to a different composition of the pane.

*APPENDIX: Tables, figures, and maps*

**Table A1 Individual timing of joining the NIDS panel**

| **Timing of joining the NIDS Panel** | **Number of individuals** |
| --- | --- |
| Wave 1 (2008) | 4,012 |
| Wave 2 (2010/2011) | 480 |
| Wave 3 (2012) | 363 |
| Wave 4 (2015/2015) | 589 |
| Wave 5 (2017/2018) | 993 |
| Total (NIDS-CRAM sample) | 6,437 |
| *Note: The table presents when individuals joined the NIDS panel study. These individuals form our analytical sample and the NIDS-CRAM study. | |

Table A2 Robustness: Difference-in-Difference and heterogeneous difference-in-difference analysis by wealth for health with covariates including chronic health condition and positive COVID-19 test

|  | (1) | (2) | (3) | (4) | (5) | (6) |
| --- | --- | --- | --- | --- | --- | --- |
|  |  | Wealth Quartiles | 1st Wealth Quartile vs Rest |  | Wealth Quartiles | 1st Wealth Quartile vs Rest |
| DD | -0.219*** |  |  | -0.207*** |  |  |
|  | (0.032) |  |  | (0.032) |  |  |
| DD 1st wealth quartile |  | -0.255*** | -0.255*** |  | -0.256*** | -0.257*** |
|  |  | (0.066) | (0.066) |  | (0.066) | (0.066) |
| DD 2nd wealth quartile |  | 0.091 |  |  | 0.112 |  |
|  |  | (0.094) |  |  | (0.094) |  |
| DD 3rd wealth quartile |  | 0.044 |  |  | 0.054 |  |
|  |  | (0.092) |  |  | (0.092) |  |
| DD 4th wealth quartile |  | 0.032 |  |  | 0.057 |  |
|  |  | (0.091) |  |  | (0.090) |  |
| DD 2nd+3rd+4th wealth quartile |  |  | 0.049 |  |  | 0.069 |
|  |  |  | (0.076) |  |  | (0.076) |
| Constant | 2.921*** | 2.892*** | 2.915*** | 2.969*** | 2.941*** | 2.964*** |
|  | (0.254) | (0.260) | (0.254) | (0.248) | (0.254) | (0.248) |
|  |  |  |  |  |  |  |
| Observations | 26,813 | 26,813 | 26,813 | 26,813 | 26,813 | 26,813 |
| Individuals | 6,285 | 6,285 | 6,285 | 6,285 | 6,285 | 6,285 |
| R-squared | 0.186 | 0.189 | 0.187 | 0.202 | 0.205 | 0.203 |
| Time effects | Yes | Yes | Yes | Yes | Yes | Yes |
| Covariates | No | No | No | Yes | Yes | Yes |
| District fixed effects | No | No | No | Yes | Yes | Yes |
| Individual fixed effects | No | No | No | No | No | No |
| F-Stat: Parallel trends | 0.332 | 0.628 | 0.190 | 0.286 | 0.635 | 0.151 |
| Prob > F: Parallel trends | 0.564 | 0.642 | 0.827 | 0.593 | 0.637 | 0.860 |
| Individual clustered standard errors in parentheses; *** p<0.01, ** p<0.05, * p<0.1; The outcome variable is individual self-rated health, with higher values indicating better health. Columns (4) to (6) include additional individual fixed effects. We control for the fully interacted difference-in-difference framework but present only the difference-in-difference estimators for each model. DD stands for difference-in-difference. As the sample size is reduced, we present in columns (1) to (3) the model without the two additional covariates but with similar sample size as comparators. | | | | | | |

Table A3 Robustness: Heterogeneous difference-in-difference analysis of cash transfer mitigation effects for the lowest wealth quartile for self-rated health covariates including chronic health condition and positive COVID-19 test

|  | (1) | (2) | (3) | (4) |
| --- | --- | --- | --- | --- |
|  | CSG: 1st Wealth Quartile | CSG Scale-up: 1st Wealth Quartile | CSG: 1st Wealth Quartile | CSG Scale-up: 1st Wealth Quartile |
| DD No CSG | -0.527*** | -0.527*** | -0.480*** | -0.481*** |
|  | (0.123) | (0.123) | (0.122) | (0.122) |
| DD CSG May 2020 scale-up |  | 0.571*** |  | 0.533** |
|  |  | (0.215) |  | (0.215) |
| DD CSG June 2020 scale-up |  | 0.305** |  | 0.252* |
|  |  | (0.151) |  | (0.150) |
| DD CSG | 0.368** |  | 0.317** |  |
|  | (0.146) |  | (0.145) |  |
| Constant | 2.510*** | 2.510*** | 2.986*** | 2.984*** |
|  | (0.083) | (0.083) | (0.241) | (0.240) |
|  |  |  |  |  |
| Observations | 6,669 | 6,669 | 6,669 | 6,669 |
| Individuals | 1,523 | 1,523 | 1,523 | 1,523 |
| R-squared | 0.119 | 0.120 | 0.224 | 0.227 |
| Time effects | Yes | Yes | Yes | Yes |
| Covariates | No | No | Yes | Yes |
| District fixed effects | No | No | Yes | Yes |
| Individual fixed effects | No | No | No | No |
| F-Stat: Parallel trends | 0.685 | 0.495 | 0.135 | 0.122 |
| Prob > F: Parallel trends | 0.504 | 0.686 | 0.874 | 0.947 |
| Individual clustered standard errors in parentheses; *** p<0.01, ** p<0.05, * p<0.1; The outcome variable is individual self-rated health, with higher values indicating better health. Column (3) and (4) include additional individual fixed effects. We control for the fully interacted difference-in-difference framework but present only the difference-in-difference estimators for each model. DD stands for difference-in-difference. As the sample size is reduced, we present in columns (1) and (2) the model without the two additional covariates but with similar sample size as comparators. | | | | |

Table A4 Parallel trends tests for CES-D and high blood pressure for all specifications

| **Model (1):**  **Centre for epidemiological studies depression scale** |  | Wealth Quartiles | 1st Wealth Quartile vs Rest | CSG:  1st Wealth Quartile | CSG Scale-up: 1st Wealth Quartile |
| --- | --- | --- | --- | --- | --- |
| F-Stat: Parallel trends | 0.849 | 0.274 | 0.434 | 0.0766 | 0.517 |
| Prob > F: Parallel trends | 0.357 | 0.895 | 0.648 | 0.926 | 0.671 |
|  |  |  |  |  |  |
| **Model (2):**  **High blood pressure** |  | Wealth Quartiles | 1st Wealth Quartile vs Rest | CSG:  1st Wealth Quartile | CSG Scale-up: 1st Wealth Quartile |
| F-Stat: Parallel trends | 2.379 | 0.737 | 1.187 | 0.248 | 0.486 |
| Prob > F: Parallel trends | 0.123 | 0.566 | 0.305 | 0.780 | 0.692 |
| Reported F-tests of significance of DD estimation and joint F-tests of significance of the heterogeneous DD estimation. The outcome in model (1) is the Centre for epidemiological depression scale; the outcome in model (2) is high blood pressure. | | | | | |

Table A5 Construct validity of self-rated health assessed by CES-D and high blood pressure

|  | (1) | (2) | (3) | (4) |
| --- | --- | --- | --- | --- |
|  | Self-rated health | Self-rated health:  Beta-coefficients | Self-rated health | Self-rated health:  Beta-coefficients |
|  |  |  |  |  |
| High blood pressure | -0.294*** | -0.122 | -0.041** | -0.017 |
|  | (0.019) |  | (0.018) |  |
| CES-D | -0.038*** | -0.157 | -0.031*** | -0.125 |
|  | (0.002) |  | (0.002) |  |
| Constant | 3.021*** |  | 3.183*** |  |
|  | (0.036) |  | (0.273) |  |
|  |  |  |  |  |
| Observations | 20,378 | 20,378 | 20,378 | 20,378 |
| Individuals | 6,342 | 6,342 | 6,342 | 6,342 |
| R-squared | 0.05 | 0.05 |  | 0.164 |
| Time effects | Yes | Yes | Yes | Yes |
| Covariates* | No | No | Yes | Yes |
| District fixed effects | No | No | Yes | Yes |
| Individual fixed effects | No | No | No | No |
| Individual clustered standard errors in parentheses; *** p<0.01, ** p<0.05, * p<0.1; The outcome variable is individual self-rated health, with higher values indicating better health. The regression is limited to the time prior the shock, i.e. before 2020, as CES-D and high blood pressure are not observed in the NIDS CRAM. *We control in columns (3) and (4) for the age group and gender of the individual. | | | | |

Table A6 Wilcoxon signed-rank tests of variations in lowest income and wealth quartiles

|  | 1. **NIDS-CRAM wave 1:**   **Rank stability of 1st Income Quartile** | 1. **NIDS wave 5:**   **Rank stability of 1st Wealth Quartile** |
| --- | --- | --- |
| **NIDS wave 5** | 0.824 |  |
| **NIDS wave 4** | 0.902 | 0.864 |
| **NIDS wave 3** | 0.302 |  |
| **NIDS wave 2** | 0.324 | 0.494 |
| **NIDS wave 1** | 0.687 |  |
| P-values from Wilcoxon signed-rank tests with the null-hypothesis of no difference in rank; comparing in model (1) rank variations in the first (lowest) income quartile defined in values from the first wave of the NIDS-CRAM versus values defined in different NIDS waves; comparing in model (2) rank variations in the first (lowest) wealth quartile in values from the 5th wave of the NIDS to values defined at the NIDS wave 4 and NIDS wave 2. | | |

Table A7 Robustness: Difference-in-Difference and heterogeneous effect difference-in-Difference analysis by wealth and cash transfer for health: using asset quartiles defined in 2014/2015 values

|  | (1) | (2) | (3) | (4) | (5) |
| --- | --- | --- | --- | --- | --- |
|  |  | Wealth Quartiles | 1st Wealth Quartile vs Rest | CSG: 1st Wealth Quartile | CSG Scale-up: 1st Wealth Quartile |
| DD | -0.247*** |  |  |  |  |
|  | (0.035) |  |  |  |  |
| DD 1st wealth quartile |  | -0.273*** | -0.273*** |  |  |
|  |  | (0.071) | (0.071) |  |  |
| DD 2nd wealth quartile |  | 0.088 |  |  |  |
|  |  | (0.099) |  |  |  |
| DD 3rd wealth quartile |  | 0.071 |  |  |  |
|  |  | (0.099) |  |  |  |
| DD 4th wealth quartile |  | -0.032 |  |  |  |
|  |  | (0.099) |  |  |  |
| DD 2nd+3rd+4th wealth quart. |  |  | 0.039 |  |  |
|  |  |  | (0.081) |  |  |
| DD No CSG |  |  |  | -0.516*** | -0.516*** |
|  |  |  |  | (0.138) | (0.138) |
| DD CSG May 2020 scale-up |  |  |  |  | 0.656*** |
|  |  |  |  |  | (0.224) |
| DD CSG June 2020 scale-up |  |  |  |  | 0.239 |
|  |  |  |  |  | (0.166) |
| DD CSG |  |  |  | 0.341** |  |
|  |  |  |  | (0.161) |  |
| Constant | 2.698*** | 2.685*** | 2.686*** | 2.644*** | 2.646*** |
|  | (0.031) | (0.038) | (0.038) | (0.076) | (0.076) |
|  |  |  |  |  |  |
| Observations | 27,053 | 27,053 | 27,053 | 6,834 | 6,834 |
| Individuals | 5,104 | 5,104 | 5,104 | 1,276 | 1,276 |
| R-squared | 0.094 | 0.097 | 0.095 | 0.112 | 0.114 |
| Time effects | Yes | Yes | Yes | Yes | Yes |
| Covariates | No | No | No | No | No |
| District fixed effects | No | No | No | No | No |
| Individual fixed effects | No | No | No | No | No |
| F-Stat: Parallel trends | 1.154 | 0.618 | 1.025 | 0.786 | 0.640 |
| Prob > F: Parallel trends | 0.283 | 0.650 | 0.359 | 0.456 | 0.589 |
| Individual clustered standard errors in parentheses; *** p<0.01, ** p<0.05, * p<0.1; The outcome variable is individual self-rated health, with higher values indicating better health. We control for the fully interacted difference-in-difference framework but present only the difference-in-difference estimators for each model. DD stands for difference-in-difference, | | | | | |

**Table A8 Robustness: Difference-in-Difference and heterogeneous difference-in-difference analysis by wealth for health using the panel from 2014 onwards**

|  | (1) | (2) | (3) |
| --- | --- | --- | --- |
|  |  | Wealth Quartiles | 1st Wealth Quartile vs Rest |
| DD | -0.227*** |  |  |
|  | (0.034) |  |  |
| DD 1^st^ wealth quartile |  | -0.261*** | -0.261*** |
|  |  | (0.068) | (0.068) |
| DD 2nd wealth quartile |  | 0.091 |  |
|  |  | (0.097) |  |
| DD 3rd wealth quartile |  | 0.043 |  |
|  |  | (0.095) |  |
| DD 4th wealth quartile |  | 0.029 |  |
|  |  | (0.094) |  |
| DD 2nd+3rd+4th wealth quartile |  |  | 0.047 |
|  |  |  | (0.078) |
| Constant | 2.822*** | 2.841*** | 2.844*** |
|  | (0.028) | (0.037) | (0.037) |
|  |  |  |  |
| Observations | 17,919 | 17,919 | 17,919 |
| Individuals | 6,437 | 6,437 | 6,437 |
| R-squared | 0.105 | 0.111 | 0.106 |
| Time effects | Yes | Yes | Yes |
| Covariates | No | No | No |
| District fixed effects | No | No | No |
| Individual fixed effects | No | No | No |
| F-Stat: Parallel trends | 0.592 | 0.486 | 0.676 |
| Prob > F: Parallel trends | 0.442 | 0.746 | 0.508 |
| Individual clustered standard errors in parentheses; *** p<0.01, ** p<0.05, * p<0.1; The outcome variable is individual self-rated health, with higher values indicating better health. The models are estimated on a reduced panel, including observations from 2014 onwards. We control for the fully interacted difference-in-difference framework but present only the difference-in-difference estimators for each model. DD stands for difference-in-difference. | | | |

**Table A9 Heterogeneous difference-in-difference analysis of cash transfer mitigation effects for the lowest wealth quartile for health using the panel from 2014 onwards**

|  | (1) | (3) |
| --- | --- | --- |
|  | CSG: 1st Wealth Quartile | CSG Scale-up: 1st Wealth Quartile |
| DD No CSG | -0.473*** | -0.473*** |
|  | (0.128) | (0.128) |
| DD CSG May 2020 scale-up |  | 0.418* |
|  |  | (0.222) |
| DD CSG June 2020 scale-up |  | 0.256 |
|  |  | (0.155) |
| DD CSG | 0.296* |  |
|  | (0.151) |  |
| Constant | 2.909*** | 2.909*** |
|  | (0.077) | (0.077) |
|  |  |  |
| Observations | 4,355 | 4,355 |
| Individuals | 1,561 | 1,561 |
| R-squared | 0.146 | 0.149 |
| Time effects | Yes | Yes |
| Covariates | No | No |
| District fixed effects | No | No |
| Individual fixed effects | No | No |
| F-Stat: Parallel trends | 0.519 | 0.457 |
| Prob > F: Parallel trends | 0.595 | 0.713 |
| Individual clustered standard errors in parentheses; *** p<0.01, ** p<0.05, * p<0.1; The outcome variable is individual self-rated health, with higher values indicating better health. The models are estimated on a reduced panel, including observations from 2014 onwards. We control for the fully interacted difference-in-difference framework but present only the difference-in-difference estimators for each model. DD stands for difference-in-difference. | | |

Figure A1 Parallel trends in age by exposure status

Figure A2 Parallel trends in household size by exposure status

Figure A3 Parallel trends in highest educational level by exposure status

Figure A4 Parallel trends in CES-D by exposure status

Figure A5 Parallel trends in high blood pressure by exposure status

| 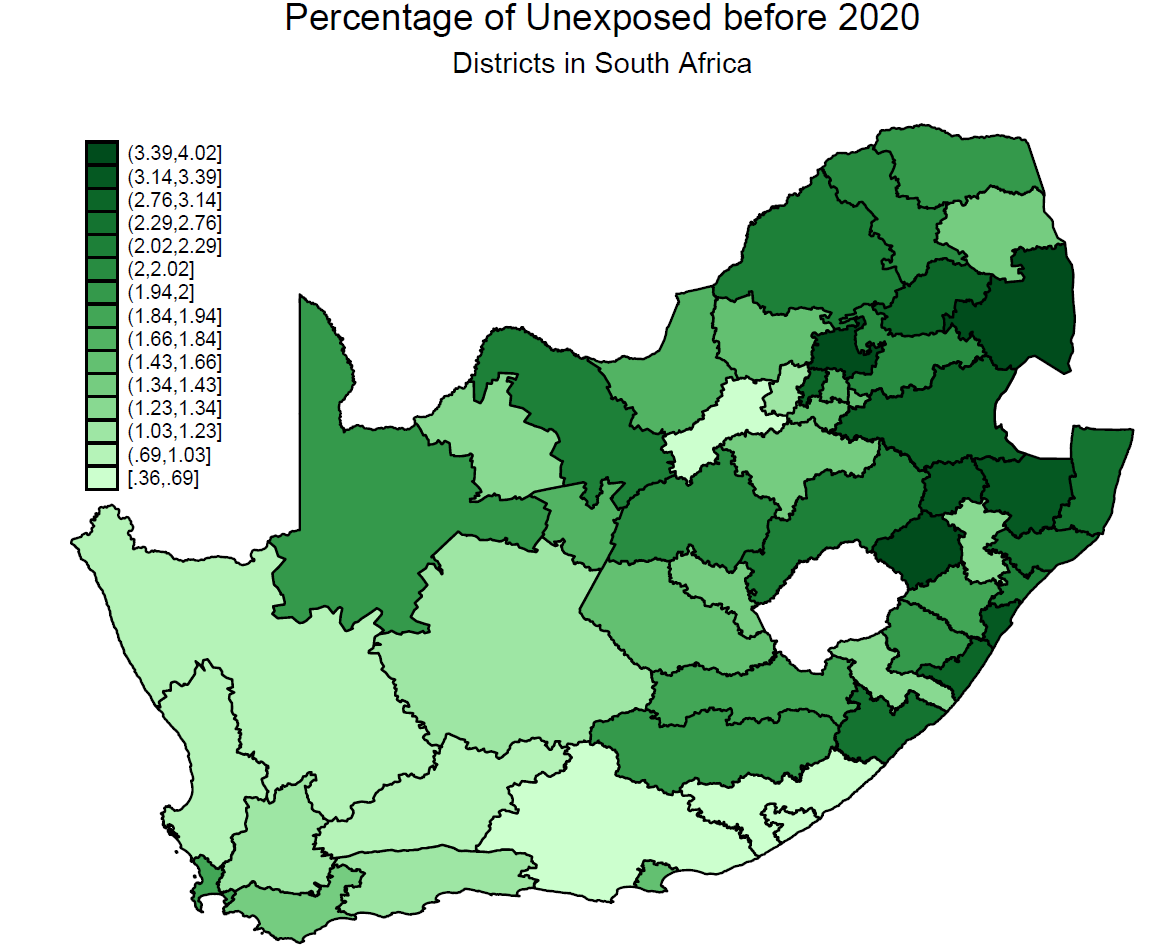 |
| --- |
| 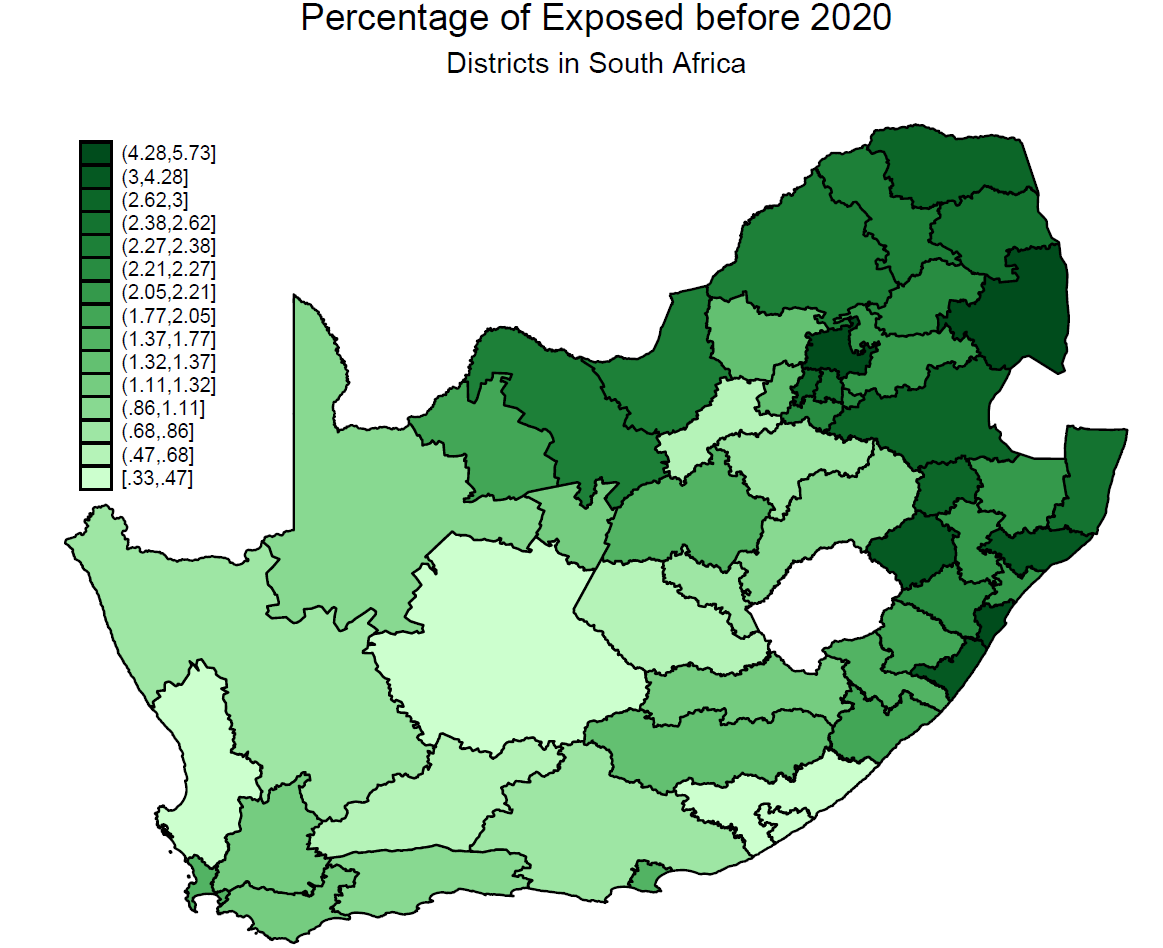 |

Map A1 Percentage concentration of exposed and unexposed individuals before 2020 (pre-shock) by South African districts, with darker shades of green indicating higher concentration.
